# Microtubule-Based Control of Motor-Clutch System Mechanics in Glioma Cell Migration

**DOI:** 10.1016/j.celrep.2018.10.101

**Published:** 2018-11-27

**Authors:** Louis S. Prahl, Patrick F. Bangasser, Lauren E. Stopfer, Mahya Hemmat, Forest M. White, Steven S. Rosenfeld, David J. Odde

**Affiliations:** 1 Department of Biomedical Engineering, University of Minnesota, Minneapolis, MN 55455, USA; 2 Physical Sciences-Oncology Center, University of Minnesota, Minneapolis, MN 55455, USA; 3 Department of Biological Engineering, Koch Institute for Integrative Cancer Research and Physical Sciences-Oncology Center, Massachusetts Institute of Technology, Cambridge, MA 02139, USA; 4 Department of Mechanical Engineering, University of Minnesota, Minneapolis, MN 55455, USA; 5 Brain Tumor and Neuro-Oncology Center and Department of Cancer Biology, Cleveland Clinic, Cleveland, OH 44195, USA; 6 Present address: Department of Medical Oncology, Mayo Clinic, Jacksonville, FL 32224, USA

## Abstract

Microtubule-targeting agents (MTAs) are widely used chemotherapy drugs capable of disrupting microtubule-dependent cellular functions, such as division and migration. We show that two clinically approved MTAs, paclitaxel and vinblastine, each suppress stiffness-sensitive migration and polarization characteristic of human glioma cells on compliant hydrogels. MTAs influence microtubule dynamics and cell traction forces by nearly opposite mechanisms, the latter of which can be explained by a combination of changes in myosin motor and adhesion clutch number. Our results support a microtubule-dependent signaling-based model for controlling traction forces through a motor-clutch mechanism, rather than microtubules directly relieving tension within F-actin and adhesions. Computational simulations of cell migration suggest that increasing protrusion number also impairs stiffness-sensitive migration, consistent with experimental MTA effects. These results provide a theoretical basis for the role of microtubules and mechanisms of MTAs in controlling cell migration.

## In Brief

Prahl et al. examine the mechanisms by which microtubule-targeting drugs inhibit glioma cell migration. They find that dynamic microtubules regulate actin-based protrusion dynamics that facilitate cell polarity and migration. Changes in net microtubule assembly alter cell traction forces via signaling-based regulation of a motor-clutch system.

## Graphical Abstract

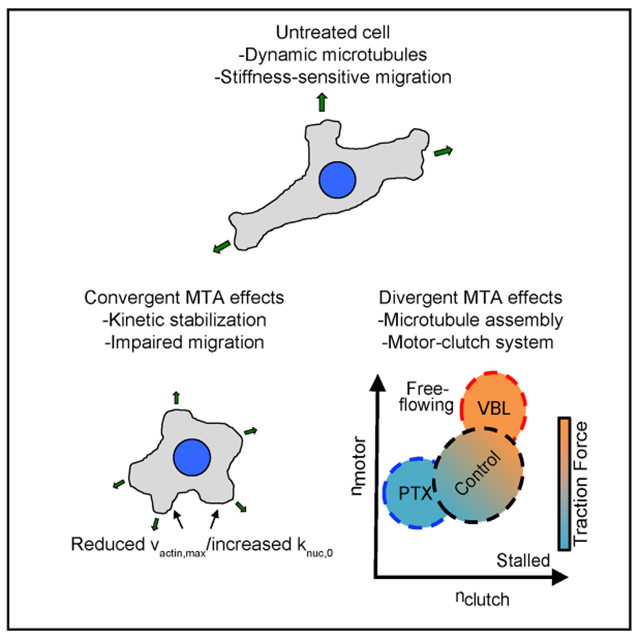

## INTRODUCTION

Extensive and rapid tumor cell proliferation and tissue invasion are hallmarks of glioblastoma (GBM, grade IV glioma) and limit patient survival and treatment efficacy (Demuth and Berens, 2004; Lefranc et al., 2005). An ideal therapeutic strategy for GBM would target both proliferating and invading cells to slow tumor dispersion (Venere et al., 2015), because slower tumor cell migration correlates with better survival outcomes (Klank et al., 2017). Dynamic microtubules are involved in both mitosis and migration and are acutely sensitive to small-molecule inhibitors, termed microtubule-targeting agents (MTAs). MTAs kinetically stabilize microtubules, which suppresses their characteristic self-assembly dynamics and interferes with their participation in cellular functions (Dumontet and Jordan, 2010). Different MTA binding sites have distinct influences on microtubule polymer assembly: taxane site-binding MTAs promote assembly, whereas MTAs that bind the *vinca* or colchicine sites promote disassembly. While assembly promoters and disassembly promoters have divergent effects on polymer assembly, their common (convergent) phenotype is kinetic stabilization (Castle et al., 2017). It has long been assumed that MTAs block cell division to stall tumor spreading, but recent work found that MTA-induced mitotic arrest is dispensable for tumor regression (Zasadil et al., 2014). This contrasting finding raises the question: is the success of MTAs in cancer therapy due to blocking tumor cell invasion?

Biophysical models of cell migration typically focus on the contributions of actin polymerization, myosin forces, and adhesion dynamics to migration. Some models also consider extracellular environmental factors, such as stiffness, which correlates with GBM aggressiveness (Miroshnikova et al., 2016). The motor-clutch model (Chan and Odde, 2008) is one such model that predicts stiffness-sensitive migration of human glioma cells (Bangasser et al., 2017; Ulrich et al., 2009). Biophysical model parameters (particularly numbers of myosin II motors and clutches) influence traction force dynamics (Bangasser et al., 2013), allowing the model to make mechanistic predictions of a wide variety of cell behaviors. However, biophysical models do not typically incorporate a role for microtubules and thus do not provide a clear mechanistic explanation for why nanomolar doses of MTAs are sufficient to influence migration of epithelial cells (Liao et al., 1995; Yang et al., 2010), endothelial cells (Bijman et al., 2006; Honoré et al., 2008; Kamath et al., 2014), neurons (Tanaka et al., 1995), glioma cells (Bergès et al., 2014; Berges et al., 2016; Pagano et al., 2012; Panopoulos et al., 2011), and other cancer cell types (Belotti et al., 1996; Jayatilaka et al., 2018).

MTAs variably affect cell traction forces (Danowski, 1989; Hui and Upadhyaya, 2017; Kraning-Rush et al., 2011; Rape et al., 2011; Stamenović et al., 2002). This may be due to MTAs disrupting microtubule-dependent adhesion turnover (Bershadsky et al., 1996; Ezratty et al., 2005; Honoré et al., 2008), or activating microtubule-based Rho GTPase signaling pathways that stimulate contractility (Chang et al., 2008; Heck et al., 2012) or protrusion (Waterman-Storer et al., 1999). Alternatively, microtubules may absorb compressive forces originating from tensions borne by F-actin and adhesions, a hypothesis that draws support from observations where traction force increases occur following microtubule depolymerization without increasing myosin II activity (Rape et al., 2011; Stamenović et al., 2002). It is unclear which of these models (e.g., signaling or mechanics) is predominantly responsible for MTA effects on cell traction and migration.

We show that paclitaxel (PTX) and vinblastine (VBL), two clinically approved MTAs, impair stiffness-sensitive glioma migration, which they each accomplish by altering actin-based protrusion dynamics. The two MTAs have distinct and divergent effects on traction forces that correlate inversely with their effects on microtubule assembly. Guided by motor-clutch model predictions, we conclude that MTAs indirectly influence motor-clutch system parameters rather than buffering against F-actin tension. Finally, we use liquid chromatography-tandem mass spectrometry (LC-MS/MS) to show that MTAs have both convergent and divergent effects on receptor tyrosine kinase (RTK) signaling networks, which correlate with effects on the motor-clutch system.

## RESULTS

### Kinetic Stabilization by MTAs Correlates with Changes in Stiffness-Sensitive Glioma Cell Migration

**Figure 1.**
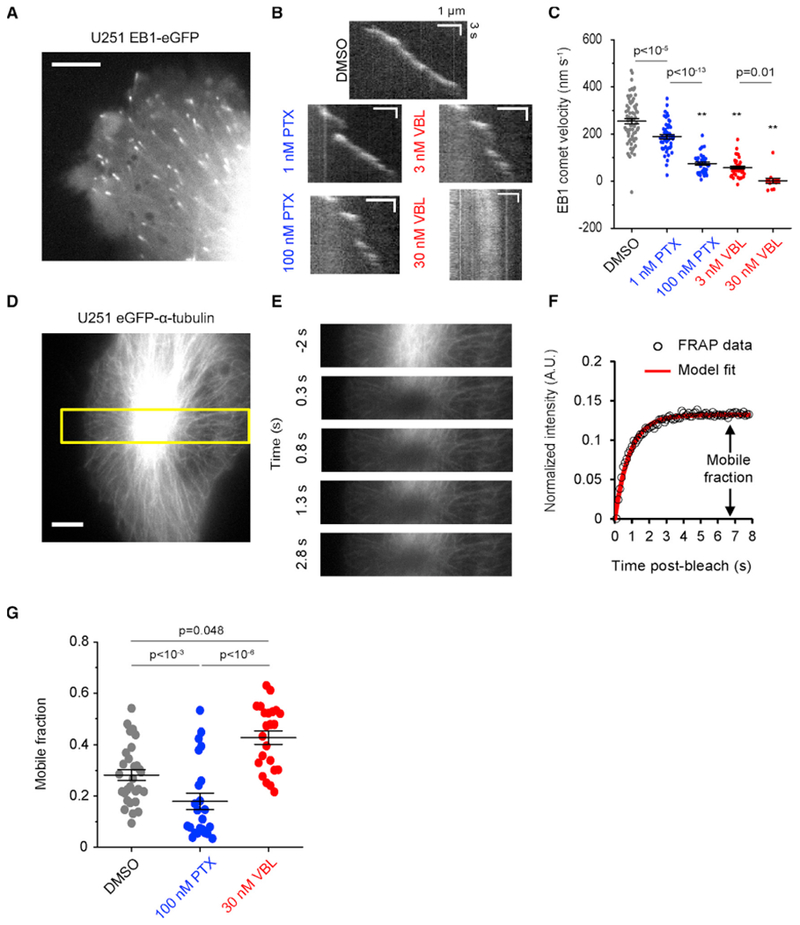
MTA Treatments that Kinetically Stabilize Microtubules Have Divergent Effects on Microtubule Assembly (A) Image of a U251 cell expressing EB1-eGFP acquired at 150× magnification. Scale, 5 μm. (B) Kymographs of EB1-eGFP in vehicle or MTA-treated cells. Horizontal scale, 1 μm; vertical scale, 3 s. (C) Measured EB1-eGFP comet velocities for the conditions in (B). Error bar sare mean ± SEM, n = 71, 49, 36, 37, 14 microtubules from N = 15, 8, 12, 9, 10 cells. p values were calculated by 1-way ANOVA with Dunn-Sidăk correction; **p < 10^−15^. Negative comet velocities indicate net negative transport during tracking due to assembly punctuated by brief shortening intervals or slow drift of kinetically stabilized plus-ends. (D) Image of a U251 cell expressing eGFP-α-tubulin acquired at 100× magnification. Scale, 5 μm. (E) Time sequence of images from the yellow box in (D). Bleaching occurs between T = −0.3 s and T = 0 s. (F) Normalized fluorescence recovery from the experiment in (D) and (E). Open circles are individual measurements; line is an exponential fit to recovered signal. (G) Quantification of tubulin mobile fraction in cells treated with vehicle or MTAs. Error bars are mean ± SEM, n = 29, 23, 23 cells. p values were calculated by Kruskal-Wallis test with Dunn-Sidăk correction.

Recent work from our laboratory shows how distinct thermodynamic and kinetic mechanisms of MTAs drive a kinetic stabilization phenotype that slows microtubule growth (Castle et al., 2017). Glioma cell migration is reportedly sensitive to nanomolar concentrations of various MTAs (Berges et al., 2014, 2016; Pagano et al., 2012; Panopoulos et al., 2011), so we sought to understand how MTAs influence microtubule dynamics in glioma cells. We transfected U251 human glioma cells with fluorescently tagged end-binding protein 1 (EB1-eGFP), which labels growing microtubule ends (Figure 1A), and treated them with either DMSO (vehicle), or varying doses (1–100 nM) of either MTA. PTX and VBL each reduced the number of visible EB1-eGFP-decorated plus-ends compared to DMSO controls (Figure S1A). Tracking EB1-eGFP signal from streaming time-lapse acquisition (Prahl et al., 2014; Seetapun et al., 2012) (Figures S1B-S1D) revealed slower growth speed with increasing MTA dose as well as near-zero speeds in 30 nM VBL-treated cells indicating stalled growth (Figures 1B and 1C). These observations are consistent with kinetic stabilization as a convergent effect of MTAs.

Castle et al. (2017) showed that 10–100 nM concentrations of PTX and VBL influence net microtubule assembly in epithelial cells. Changes in microtubule assembly could affect signaling pathways related to migration (Heck et al., 2012) or directly influence cell mechanical properties (Stamenović et al., 2002), so we sought to measure microtubule assembly in MTA-treated U251 cells expressing eGFP-α-tubulin (Figure 1D). We used fluorescence recovery after photobleaching (FRAP), as described previously (Figure 1E) (Castle et al., 2017), to estimate the mobile fraction from recovered signal (Figure 1F). PTX (assembly promoter) decreased the mobile fraction, while VBL (disassembly promoter) increased the mobile fraction (Figure 1G), corresponding to an increase or decrease in immobile fraction, respectively. Our FRAP results indicate that the two MTAs divergently affect microtubule assembly in U251 cells.

**Figure 2.**
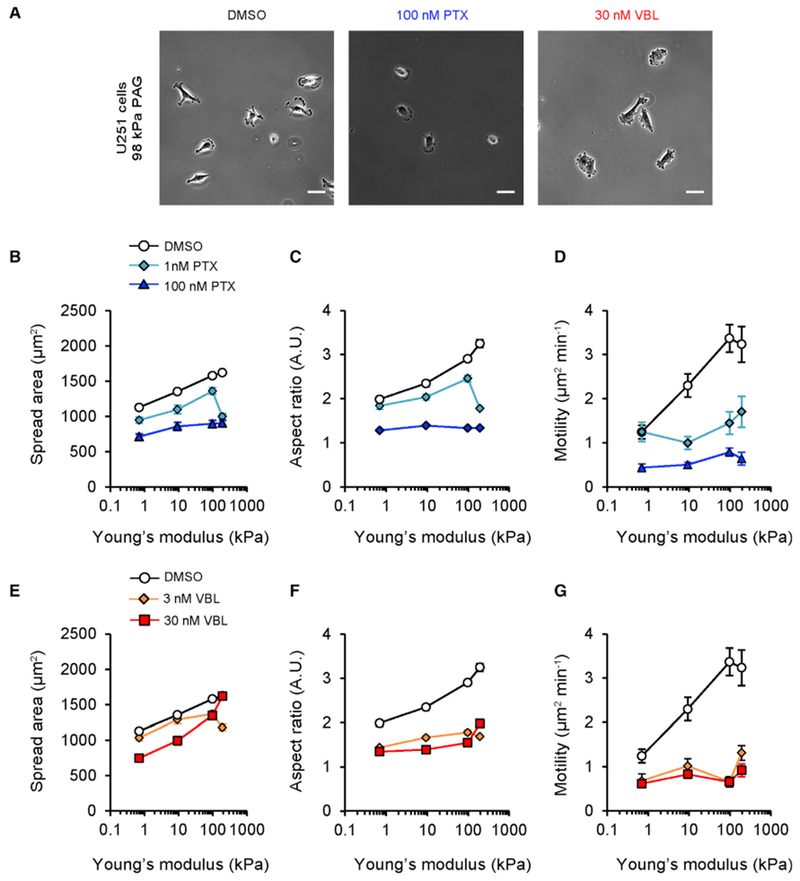
MTAs Disrupt Stiffness-Sensitive Glioma Cell Spreading, Polarization, and Migration (A) Images of U251 cells on 98 kPa PAGs acquired at 10× magnification using phase contrast optics. Cells were treated with vehicle (DMSO), 100 nM PTX, or 30 nM VBL. Scale, 50 μm. (B–D) Spread area (B), aspect ratio (C), or motility (D) as a function of PAG Young’s modulus for U251 cells treated with vehicle (DMSO) or 1–100 nM PTX; DMSO: n = 130, 107, 137, 72 cells; 1 nM PTX: n = 51, 74, 53, 41 cells; 100 nM PTX: n = 83, 94, 91, 37 cells. (E–G) Spread area (E), aspect ratio (F), or motility (G) as a function of PAG Young’s modulus for U251 cells treated with vehicle (DMSO) or 3–30 nM VBL. Vehicle (DMSO) data are reproduced from (B)–(D); 3 nM VBL: n = 47, 62, 54, 43 cells; 30 nM VBL: n = 78, 119, 99, 45 cells. Data are represented as mean ± SEM; pairwise statistical comparisons are reported in Table S1.

Next, we sought to correlate changes in microtubule dynamics caused by MTAs with migration. Previous studies found that U251 cells exhibit stiffness-sensitive polarization and migration on compliant polyacrylamide gels (PAGs) with fastest migration on 100 kPa PAGs (Bangasser et al., 2017; Ulrich et al., 2009). This result is consistent with a recently described motor-clutch model of cell migration (Bangasser et al., 2017), so we reasoned that experimental migration measurements could be used to test MTA mechanisms using a modeling-based approach. Spread area, aspect ratio, and motility of individual cells were measured in the presence of vehicle or MTAs on PAGs functionalized with type I collagen (0.7–195 kPa Young’s modulus) (Figure 2A; Video S1). Vehicle control measurements resembled the previously described trends in spread area, aspect ratio, and motility with increasing PAG modulus (Figures 2B–2G) (Bangasser et al., 2017; Ulrich et al., 2009). MTAs significantly reduced motility and aspect ratio in a dose-dependent fashion (Figures 2B–2G), reflecting the loss of long protrusions and rounded morphologies of MTA-treated cells (Figure 2A; Video S1). Interestingly, either drug almost completely suppressed stiffness-sensitive migration, suggesting that MTAs influence the motor-clutch system in glioma cells.

### MTAs Have Divergent Effects on Cell Traction and Actin Flow as Predicted by a Motor-Clutch Model

**Figure 3.**
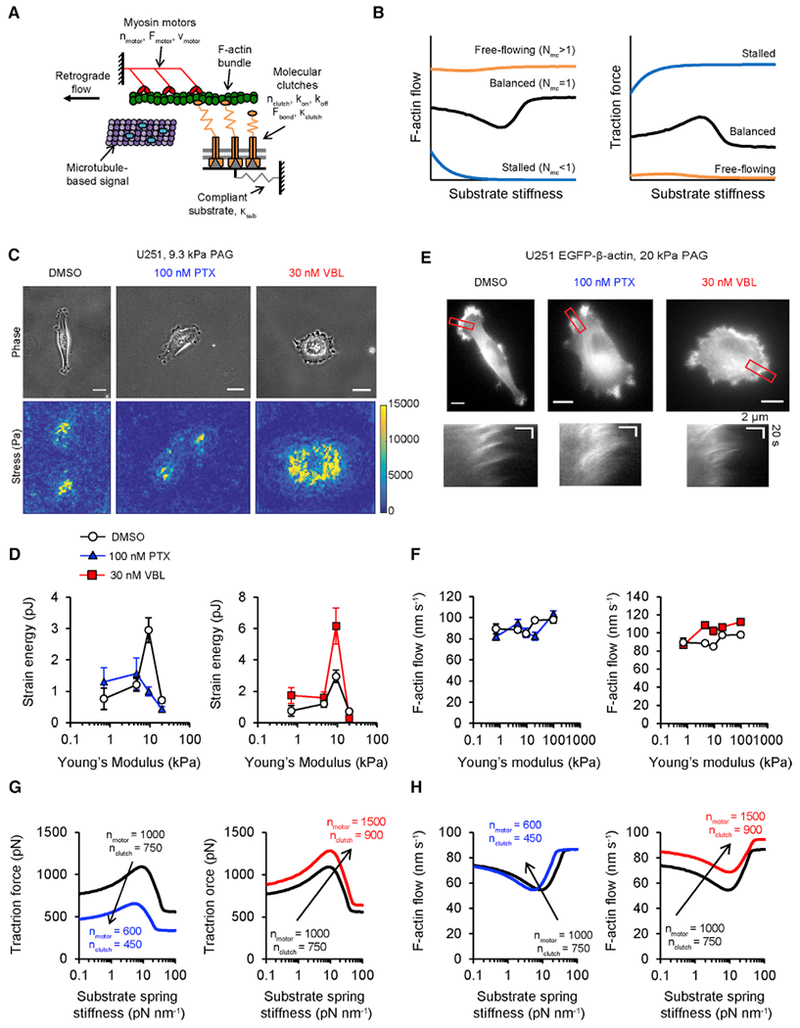
MTAs Have Divergent Effects on Traction Strain Energy and F-Actin Flow Speed, Consistent with Motor-Clutch Model Predictions (A) Motor-clutch model schematic that includes putative signaling role of microtubules. Myosin II motors (red) cause retrograde flow in an F-actin bundle (green). Molecular clutches (orange) connect F-actin to a compliant substrate (gray). Microtubules (purple) transport signaling factors (cyan) that regulate components of the motor-clutch system. (B) Motor-clutch model predictions of traction force and F-actin flow sped for balanced (N_mc_ = 1), free-flowing (N_mc_ > 1), and stalled (N_mc_ < 1) simulations. (C) Phase images (top row) of U251 cells on 9.3 kPa PAGs containing 0.2 μm fluorescent microspheres acquired at 60× magnification. Cells were treated with vehicle (DMSO), 100 nM PTX, or 30 nM VBL. Traction stress (bottom row) is calculated for each cell using a previously described method (Bangasser et al., 2017; Butler et al., 2002). Scale, 20 μm. (D) Strain energy measured on 0.7–20 kPa PAGs for the conditions in (C). DMSO measurements are repeated in both plots; DMSO: n = 47, 103, 184, 87 cells; 100 nM PTX: n = 38, 47, 84, 53 cells; 30 nM VBL: n = 35, 79, 100, 59 cells. Data are represented as mean ± SEM; pairwise statistical comparisons are reported in Table S1. (E) U251 cells expressing eGFP-α-actin on 20 kPa PAGs (top row) acquired at 60× magnification. Treatment conditions are the same as those used in (C). Kymographs (bottom row) of F-actin flow acquired on the locations marked in the corresponding image. Scale, 10 μm. (F) F-actin flow measured on 0.7–195 kPa PAGs for the conditions in (E). DMSO measurements are repeated in both plots; DMSO: n = 33, 29, 36, 31, 25 cells; 100 nM PTX: n = 23, 27, 25, 30, 17 cells; 30 nM VBL: n = 16, 19, 20, 21, 20 cells. Data are represented as mean ± SEM, pairwise statistical comparisons are reported in Table S1. (G) Motor-clutch model predictions of traction force for parameter sets representing DMSO (black), PTX(blue), or VBL(red) treatments. DMSO output is repeated in both plots as a reference. (H) Motor-clutch model predictions of F-actin flow for the parameter sets in (G). DMSO output is repeated in both plots as a reference. Other simulation parameters are in Methods S1.

A motor-clutch model (Figure 3A) (Chan and Odde, 2008) predicts stiffness-sensitive glioma cell behaviors, including traction force and migration (Bangasser et al., 2017). In the model, myosin II motors (n_motor_) generate F-actin retrograde flow, while dynamic binding and unbinding of molecular clutches (n_clutch_) transmits force through F-actin to a compliant substrate. Here, we focus on the hypothesis that dynamic microtubules regulate n_motor_ and n_clutch_ based on observations that MTA treatments can promote increased phosphorylated myosin regulatory light chain (pMLC) levels (Chang et al., 2008; Kolodney and Elson, 1995) or cause changes focal adhesion size (Bershadsky et al., 1996; Ezratty et al., 2005), reflecting accumulation or loss of clutches. Previous model characterization (Bangasser et al., 2013) shows that stiffness sensitivity is especially sensitive to the ratio of n_motor_ to n_clutch_, which we define in Equation 1:

(Equation 1) $$N_{mc}=\frac{n_{motor}}{n_{clutch}}.$$

Notably, n_motor_ and n_clutc*h*_ need to be approximately equal (N_mc_ ≈ 1, balanced motors and clutches in Figure 3B) to produce both stiffness-sensitive traction forces and non-zero F-actin retrograde flows. In stalled (N_mc_ < 1) or free-flowing (N_mc_ > 1) cells, the model predicts stiffness insensitivity (uniform traction forces and F-actin flow speed and loss of the optimum; Figure 3B). These trends provide experimentally testable hypotheses for how MTAs could impair stiffness sensitivity of migration.

To test whether MTAs affect U251 cell traction, we used traction force microscopy (TFM) to measure strain energy in cells treated with vehicle, 100 nM PTX, or 30 nM VBL (Figure 3C). The vehicle-treated group generated maximum strain energy on 9.3 kPa PAGs, consistent with prior reports (Bangasser et al., 2017). Cells treated with 100 nM PTX generated maximum strain energy on 4.6 kPa PAGs, which is softer than the optimum for vehicle-treated cells (Figure 3D), while the VBL group generated maximum strain energy on 9.3 kPa PAGs (Figure 3D). For cells treated with VBL, mean strain energy on substrates softer than 20 kPa were statistically similar to vehicle (Table S1). To measure F-actin flow under the same conditions, we obtained time-lapse videos of U251 cells transfected with eGFP-β-actin (Figure 3E; Video S2). Tracking flow features at the cell edge revealed stiffness-sensitive F-actin flow speed in vehicle-treated cells (Figure 3F). We did not observe a minimum flow speed on 10-100 kPa substrates like in previous reports (Bangasser et al., 2017), although fast actin flow speeds on stiff PAGs correlate with low strain energy, a key model prediction (Chan and Odde, 2008). F-actin flow speeds were similar between vehicle and PTX groups, with the exception of a significant decrease at 20 kPa, whereas VBL significantly increased flow speed on 4.6–9.3 kPa PAGs (Figure 3F; Table S1). Altogether, these data suggest that MTAs affect motor-clutch system properties, but do not eliminate stiffness sensitivity through the mechanisms outlined in Figure 3B.

Next, we tested whether the motor-clutch model could recapitulate experimentally observed traction force and F-actin flow speed in predictable ways. We simulated vehicle-treated cells with N_mc_ = 1.33 to better match measured F-actin flow speeds while retaining stiffness-sensitive traction force (Figures 3G and 3H). Reducing both n_motor_ and n_clutch_ decreased the total traction force output, consistent with decreased strain energy in PTX-treated cells (N_mc_ = 1.33 for PTX, because F-actin flow speeds were similar to control) (Figure 3F). To simulate the effects of VBL, we increased n_motor_ and n_clutch_ (and N_mc_ = 1.66), which increased both traction forces and F-actin flow speed (Figures 3G and 3H). We conclude that modest changes in n_motor_ and n_clutch_ can explain motor-clutch system changes in MTA-treated cells.

We hypothesized that the divergent effects of PTX and VBL on traction force and actin flow are due to varying microtubule polymer assembly, rather than kinetic stabilization. If this is true, MTA effects should be dose-dependent and similar to controls in the ~1 nM dose range, where changes in polymer assembly are minimal (Castle et al., 2017). To test this prediction, we measured strain energy for U251 cells on 9.3 kPa PAGs as a function of MTA dose. We observed dose-dependent linear trends with increasing MTA dose for both PTX and VBL (decreasing and increasing, respectively), with the lowest doses (1 nM PTX and 3 nM VBL) having similar mean values to vehicle-treated cells (Figures S3A and S3B). Next, we reasoned that MTAs with similar effects on microtubule polymer assembly would similarly affect strain energy. MTA effects on microtubule assembly tend to be binding site-specific (e.g., taxane site-binding drugs are assembly promoters, while *vinca* site-binding drugs are disassembly promoters), so we expected these other MTAs would have similar divergent effects. We treated U251 cells on 9.3 kPa PAGs with either epothilone B (EPB; taxane site) or vincristine (VCR; *vinca* site) and measured strain energy using TFM. Similar to PTX, strain energy of EPB-treated cells decreased with increasing dose (Figure S3C). VCR elicited a dose-dependent increasing strain energy trend (Figure S3D), similar to VBL. These results suggest that n_motor_ and n_clutch_ correlate with changes in microtubule polymer assembly.

### Motor-Clutch Model Predicts Myosin-II-Dependent Effects of MTAs

Our analysis using the motor-clutch model reveals that increased n_motor_ is consistent with increased F-actin flow speed in VBL-treated cells. Other reports indicate that disassembly-promoting MTAs increase pMLC, while assembly promoters do not (Chang et al., 2008; Kolodney and Elson, 1995). One possible explanation for the discrepancy is that microtubule-binding guanine nucleotide exchange factors (GEFs) activate upon microtubule depolymerization, in turn stimulating pMLC levels through the RhoA pathway (Chang et al., 2008; Heck et al., 2012).

**Figure 4.**
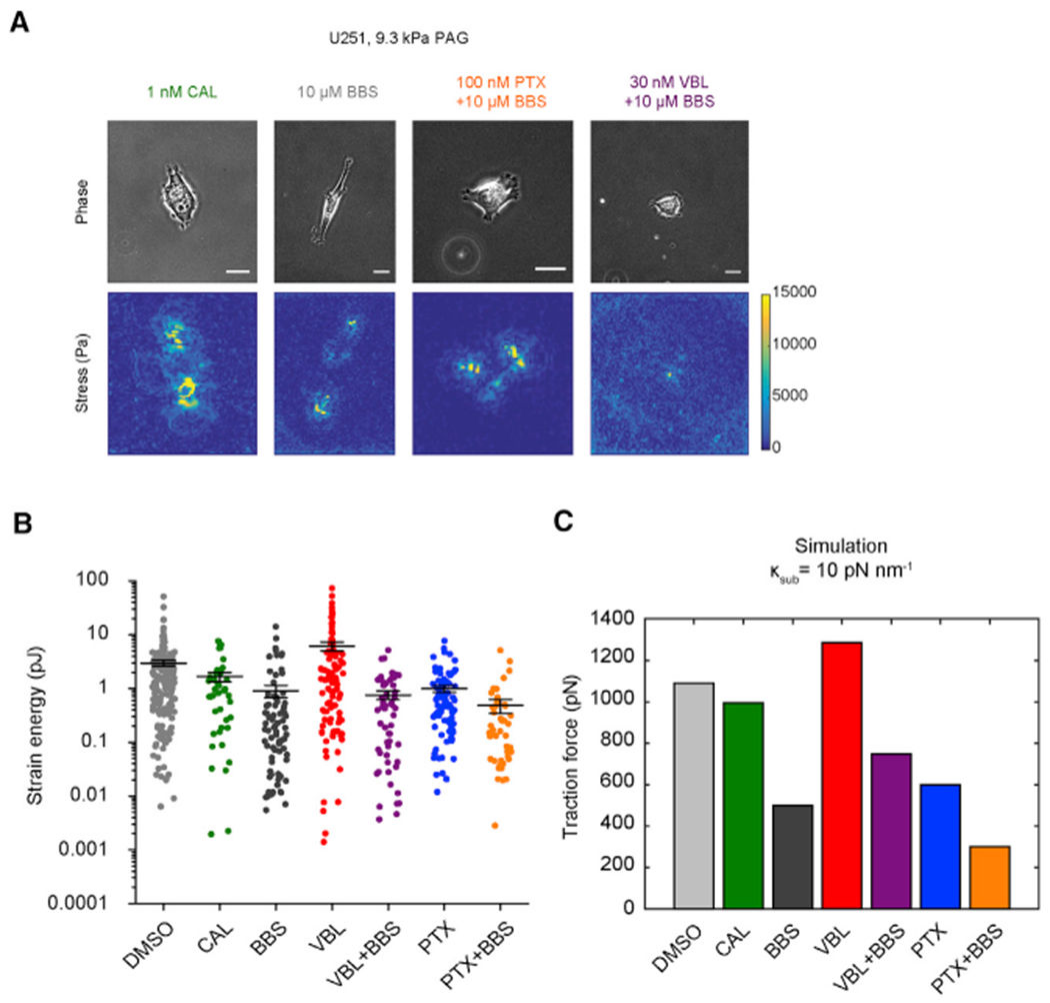
Motor-Clutch Model Predicts Traction Forces of U251 Cells Treated with MTAs or Drugs Targeting Myosin II Activity (A) Phase contrast images (top row) of U251 cells on 9.3 kPa PAGs containing 0.2 μm fluorescent microspheres acquired at 60× magnification. Conditions shown are 1 nM CAL, 10 μM BBS, 30 nM VBL, and 10 μM BBS, and 100 nM PTX and 10 μM BBS. Scale, 20 μm. Traction stress (bottom row) plots for each cell are obtained like in Figure 3C. (B) Strain energy measurements on 9.3 kPa PAGs for cells treated with DMSO, 1 nM CAL, 10 μM BBS, 30 nM VBL, 30 nM VBL, and 10 μM BBS, 100 nM PTX, or 100 nM PTX and 10 μM BBS. Bars are mean ± SEM; n = 184, 43, 81, 100, 54, 84, 45 cells; pairwise statistics are reported in Table S3. Vehicle (DMSO), 30 nM VBL, and 100 nM PTX measurements are reproduced from the 9.3 kPa data in Figure 3D. (C) Motor-clutch model-predicted traction force for simulations run at κ_sub_ = 10 pN nm^−1^. Values of n_motor_/n_clutch_ for each condition: DMSO – 1,000/750; CAL – 3,000/750; BBS – 250/750; VBL – 1,500/900; VBL+BBS – 375/900; PTX – 600/450; PTX+BBS – 150/450. Other simulation parameters are in STAR Methods.

Calyculin A (CAL) is a small molecule phosphatase inhibitor, and treatments of CAL result in increased pMLC (Chartier et al., 1991). We treated U251 cells on 9.3 kPa PAGs with 1 nM CAL and measured strain energy by TFM (Figures 4A and 4B). We observed that strain energy of CAL-treated cells decreased ~2-fold (Figure 4B), although the pairwise test was not significant (p = 0.76; Table S2). However, CAL significantly decreased strain energy compared to 30nM VBL (p < 10^−3^, Kruskal-Wallis test with Dunn-Sidak correction; Table S2). Intriguingly, we found the motor-clutch model also predicted decreasing traction force when n_motor_ is independently increased (Figure 4C). Oria et al. noted that CAL reduced adhesion size in fibroblasts, consistent with model-predictions, suggesting that increasing myosin II activity causes cells to feel substrates as if they were stiffer (Oria et al., 2017). For other cell types where CAL increases traction force (Hui and Upadhyaya, 2017; Kraning-Rush et al., 2011), increasing n_motor_ could increase traction forces in cells where the “untreated” value of n_clutch_ would be larger than n_motor_ (i.e., N_mc_ < 1; Figure S4), or by activating force-feedback adhesion reinforcement (Elosegui-Artola et al., 2016). These data support our hypothesis that VBL increases both n_motor_ and n_clutch_ in U251 cells.

Myosin II inhibition reduces glioma cell traction force and increases spreading (Bangasser et al., 2017; Ulrich et al., 2009), so we hypothesized that concurrent treatment with blebbistatin (BBS) and MTAs would reverse or override the effects of MTAs on traction and spreading. Treating cells with BBS alone decreased traction force (Figure 4B), consistent with earlier reports (Bangasser et al., 2017). Concurrent treatment with 30 nM VBL (VBL + BBS) reduced strain energy compared to 30 nM VBL alone (Figure 4B), while concomitant addition with 100 nM PTX (PTX+BBS) did not show any significant change from either drug alone (Figure 4B). BBS treatments also mitigated the decrease in spread area that follows MTA treatment (Figure S3). We were able to reproduce traction force trends in the model by changing n_motor_ and n_clutch_ to various ratios as reasonable estimates for the drug effects (Figure 4C). Parameter changes that reproduce experimental trends for MTAs, CAL, and BBS are further outlined in Figures S4A–S4C.

### Microtubule Mechanical Reinforcement Predicts Effects on F-Actin Flow but Fails to Predict Traction Effects of MTAs

**Figure 5.**
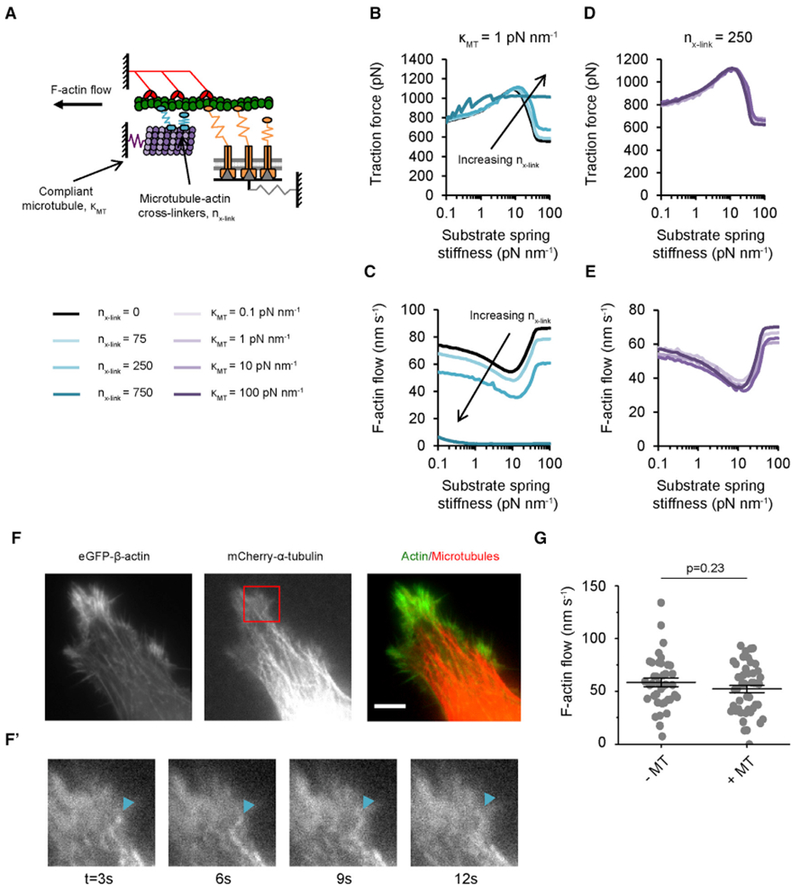
Mechanical Reinforcement Model Predictions and Observations of Microtubule-F-Actin Mechanical Interaction in Glioma Cells (A) Motor-clutch model schematic that incorporates a hypothetical mechanical interaction between microtubules and F-actin. A compliant microtubule bundle (purple) binds the F-actin bundle through dynamic cross-linkers (cyan) that function as clutches. Forces on the microtubule and substrate both resist the total myosin II motor force of the cell. (B and C) Model-predicted traction force (B) and F-actin flow (C) when n_x-link_ is varied as an independent parameter and κ_MT_ = 1 pN nm^−1^. Black represents a reference condition when n_x-link_ = 0, cyan lines represent n_x-link_ = 75, 250, and 750. (D and E) Model-predicted traction force (D) and F-actin flow (E) when κ_MT_ is varied as an independent parameter and n_x-link_ = 250. Purple lines represent κ_MT_ = 0.1–100 pN nm^−1^. Other simulation parameters are in STAR Methods. (F and F’) Images of a U251 cell expressing GFP-β-actin (green) and mCherry-α-tubulin (red) acquired at 100× magnification. Scale, 5 μm. Inset from the red box in (F) shows a microtubule plus-end bending at the periphery, indicating it is subject to compressive forces (Bicek et al., 2009; Brangwynne et al., 2006). (G) F-actin flow speed in regions where actin flow coincided with microtubule polymer (+MT) compared to regions where no microtubule was present (−MT). Error bars are mean ± SEM, data are pooled from n = 24 cells, two biological replicates, Mann-Whitney U test.

Although our results thus far support a model where MTAs work by altering n_motor_ and n_clutch_ through signaling networks, a long-standing hypothesis for MTA effects involves direct mechanical interaction between pre-stressed microtubules and F-actin structures (Stamenović et al., 2002). Microtubules bear compressive forces that resist tension on F-actin, adhesions, and the substrate, while altering microtubule polymer assembly could redistribute forces between the structures (Danowski, 1989; Stamenović et al., 2002). As this mechanical model would intuitively predict, changes in microtubule polymer assembly in U251 cells are inversely correlated with strain energy (compare Figures 1G and S2). However, the mechanical load shifting hypothesis is controversial and has often been proposed alongside alternative signaling-based explanations for MTA effects (Danowski, 1989; Rape et al., 2011). To further test a mechanical role of microtubules, we modified the motor-clutch model (Figure 5A) to include a compliant microtubule spring that can bear myosin II-based forces. Microtubules directly couple to F-actin through dynamic cross-linkers that function as clutches and bind both filaments; examples of previously characterized cross-linkers are proteins, such as ACF7 (Wu et al., 2008). We modeled the microtubule cytoskeleton as a compliant elastic spring with a stiffness of 1 pN nm^−1^ estimated from flexural rigidity measurements of purified tubulin (reviewed in VanBuren et al. [2005]).

We first used the number of cross-linkers (n_x-link_) as a proxy for polymer assembly (e.g., decreasing n_x-link_ reflects a reduction in the polymer mass and vice versa). Increasing n_x-link_ while holding other parameters fixed (Methods S1) had little effect on traction force, except when n_x-link_ = n_clutch_ = 750, which stalled actin flow (Figure 5B). Increasing n_x-link_ increased the force on the microtubule spring (Figure S4D), indicating that a portion of the motor force is transferred to each of the two springs. Next, we reasoned that changes in microtubule spring stiffness (κ_MT_) could reflect increased stiffness through either microtubule bundling (Soheilypour et al., 2015) or rigidity changes due to MTAs or microtubule-associated proteins (MAPs) (Hawkins et al., 2013). Surprisingly, changes in κ_MT_ (for a constant n_x-link_) had little effect on traction force and F-actin flow speed (Figure 5C), and microtubule force (Figure S4E). We also found that changes in n_motor_ and n_clutch_ could be combined with mechanical reinforcement to reproduce effects on traction and F-actin flow (Figures S4F–S4K). These combined results suggest that a microtubule mechanical reinforcement model does not explain MTA effects on traction force alone.

Microtubules can bear large compressive loads due to reinforcement from the actin cytoskeleton, as evident from observations of microtubule buckling (Brangwynne et al., 2006). The mechanical reinforcement model predicts that weakening cross-linking (by reducing n_x-link_) increases F-actin flow speed, as we observe with VBL (Figure 3F). However, it also predicts that strengthening cross-linking (increasing n_x-link_, as with PTX) would slow F-actin flows, which was not observed. We reasoned that if microtubules resist F-actin in U251 cells, experimentally, F-actin flow speed would decrease in the presence of microtubules, which would appear buckled. To test this, we co-transfected U251 cells with mCherry-α-tubulin and eGFP-β-actin (Figure 5D) and analyzed regions containing both flowing F-actin and microtubules, or F-actin in the absence of microtubule polymer. Instances of microtubule buckling occurred at the edge of the cell (Figure 5E; Video S3), as well as co-transport of microtubules with F-actin flows, as previously noted (Gupton et al., 2002; Waterman-Storer and Salmon, 1997). F-actin flow speed did not decrease in regions containing microtubules compared to F-actin flows that did not cross microtubules (Figure 5F; note that F-actin flows for U251 cells on glass are slower than on PAGs in Figure 3F). Based on these observations, and the lack of significant changes in model-predicted traction force (Figures 5B and 5C), we conclude that mechanical reinforcement does not fully explain our experimental results.

### Cell Migration Simulations Predict that MTA Mechanisms Include Effects on Actin Polymerization and Nucleation

**Figure 6.**
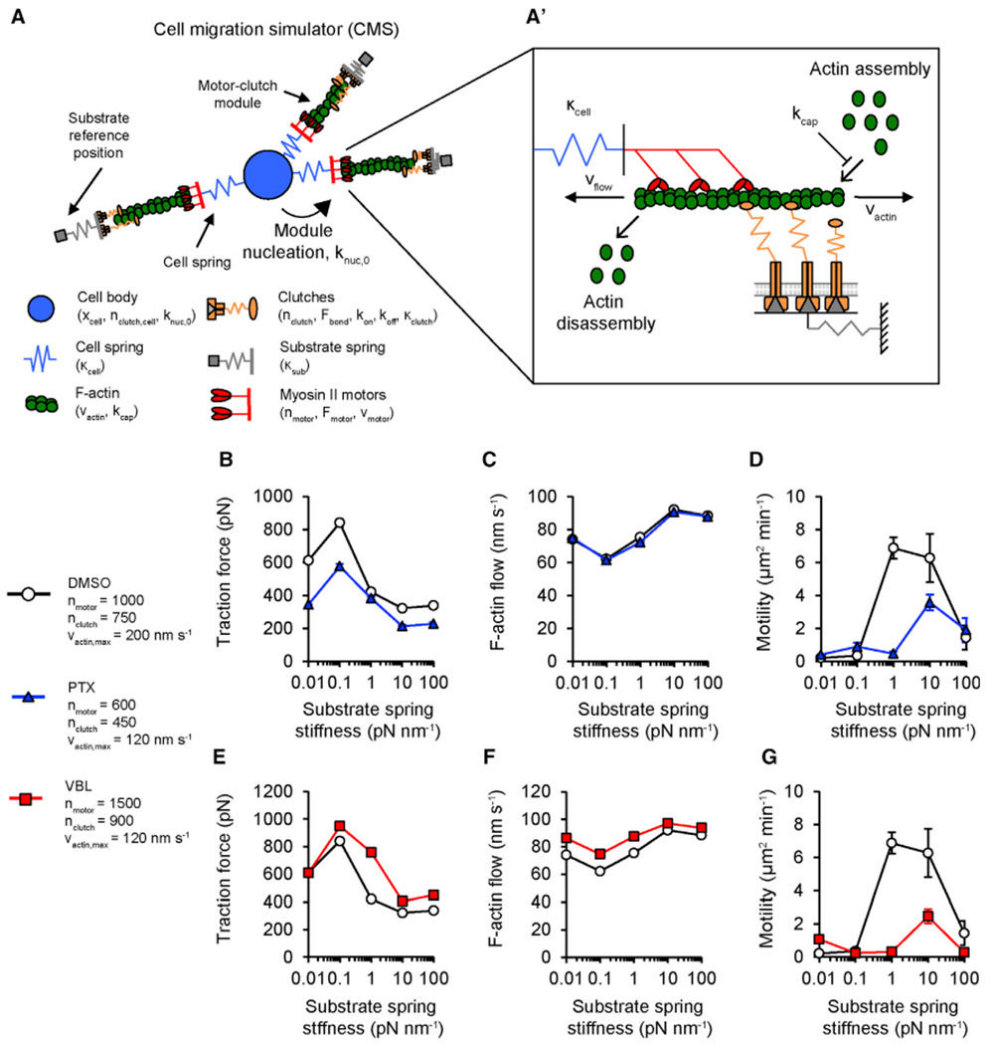
Stiffness-Sensitive Simulated Cell Migration Is Sensitive to Changes in Actin Polymerization and Nucleation Rates (A and A’) Schematic for a CMS as previously described in Bangasser et al. (2017) and Klank et al. (2017). Individual protrusion modules function as copies of the motor-clutch model described in Chan and Odde (2008) and connect to a central cell body/nucleus through cell springs (blue). New modules are nucleated at a rate (k_nuc_) that scales with the available G-actin pool. Modules extend through actin polymerization (V_actin_) that scales a maximal polymerization velocity (V_actin,max_) by the available G-actin pool. Modules shorten by F-actin retrograde flow (v_flow_), which is governed by clutch dynamics. Module capping (k_cap_) stochastically terminates actin polymerization to facilitate module turnover and cell polarization. (B–D) CMS predictions of traction force (B), F-actin flow (C), and motility (D) for a reference parameter set representing DMSO (white circles; n = 14, 8, 36, 48, 24 runs) or a PTX parameter set (blue triangles; n = 8, 16, 16, 28, 12 runs). (E–G) CMS predictions of traction force (E), F-actin flow (F), and motility (G) for reference parameters (repeated from B–D) or a VBL parameter set (red squares; n = 4, 8, 20, 24, 12 runs). Reference parameter results are repeated from (B) to (D). Reference parameter simulation data are the same as that used in Figures S5 and S6. For other parameters, see Methods S1. Data are represented as mean ± SEM, pairwise statistical comparisons between test groups and reference conditions are reported in Table S3.

While a motor-clutch model captures traction force and F-actin flow in MTA-treated cells (Figure 3), it does not directly predict how these relate to migration. To test whether these changes to the motor-clutch system were sufficient to produce stiffness insensitive migration of MTA-treated cells, we used a previously described cell migration simulator (CMS) based on the motor-clutch model (Bangasser et al., 2017; Klank et al., 2017). The CMS links motor-clutch protrusion modules to a central cell body by elastic springs representing the cell’s internal nucleo-cytoskeletal compliance (Figure 6A). Modules extend through actin polymerization, are created and destroyed stochastically, and are subject to a mass balance on total actin length (Figure 6A’). Force balances update the cell position (x_cell_) as the simulation progresses, enabling statistics of cell shape and motility from individual trajectories. A previously published parameter set (see Methods S1 for parameter values) reproduces experimental traction force, F-actin flow, and migration in U251 cells (Bangasser et al., 2017) (Figures 6B–6G), so we used this as a reference condition.

We sought to identify parameters that can reproduce the effects of MTAs on motility. First, we asked whether changing n_motor_ and n_clutch_ would cause stiffness insensitive motility observed in experiments, since these parameters matched experimental trends in strain energy and F-actin flow speed (Figure 3). Interestingly, when n_motor_ and n_clutch_ were changed to reflect the effects of either MTA, motility remained similar to the reference parameters (Figures S5A and S5B). This suggests that MTA effects on the motor-clutch system alone are not sufficient to produce the experimentally observed changes in motility.

Given these results, we next asked which other CMS parameters could replicate the effects of MTAs. Experimentally, MTA-treated cells appear to have numerous short protrusions ringing the cell body, rather than the long protrusions seen in vehicle-treated cells (Figure 2A). Additionally, reduced polarity correlates with impaired migration (Figure 2). We hypothesized that three parameter changes could each individually replicate this effect: (1) reducing the maximum actin polymerization rate (v_actin,max_); (2) increasing the module capping rate (k_cap_) that stochastically terminates actin polymerization; or (3) increasing the maximum module nucleation rate (k_nuc,0_). Reducing v_actin,max_ from 200 nm s^−1^ to 120 nm s^−1^ slowed motility, corresponding with an increase in mean module number and decreased aspect ratio on 1–100 pN nm^−1^ substrates (Figure S5C; Video S4). Similar effects are noted with increasing k_nuc_ 10-fold (Figure S5D; Video S4). Intriguingly, increasing k_cap_ 10-fold from its base value had little effect on motility, aspect ratio, or module number (Figure S5E; Video S4), suggesting motility is only weakly sensitive to increasing k_cap_. When we combined reducing v_actin,max_ with the MTA-specific changes to n_motor_ and n_clutch_, these three parameters were sufficient to reproduce experimental trends in traction force, F-actin flow, and motility seen in cells treated with PTX (Figures 6B–6D) or VBL (Figures 6E–6G). We note similar effects when k_nuc,0_ is increased instead of reducing v_actin,max_ (Figure S6). Coordination between microtubules and F-actin regulates protrusion dynamics in fibroblasts (Waterman-Storer et al., 1999), neurons (Winans et al., 2016), and fibrosarcoma cells (Jayatilaka et al., 2018), so we suggest that similar microtubule-dependent signaling relays could be responsible for controlling F-actin assembly in glioma cells.

### Divergent Effects of MTAs on Tyrosine Phosphorylation in Glioma Cells

Our results support a model where MTAs have both convergent (shape and migration) and divergent (traction forces and F-actin flow) effects on cell migration mechanics. As our results argue against a purely mechanical explanation, we hypothesized that (1) MTAs disrupt microtubule-based signaling pathways and (2) signaling network analysis would reveal convergent and divergent changes, corresponding to pathways that control F-actin polymerization and motor-clutch mechanics, respectively. Tyrosine phosphorylation (pY)-mediated signaling at focal adhesions is differentially affected by assembly and disassembly-promoting MTAs (Bershadsky et al., 1996), so we sought to investigate the involvement of other pY-mediated signaling pathways in response to MTAs.

**Figure 7.**
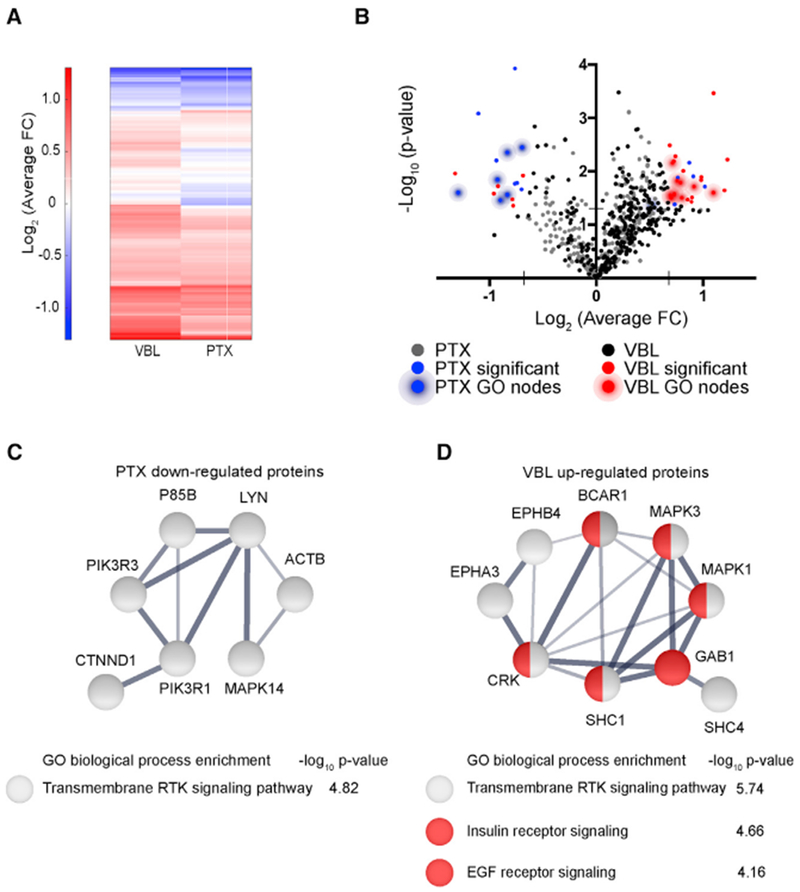
Quantitative Analysis of pY-Mediated Signaling Pathways in MTA-Treated Cells (A) Heatmap of log_2_-transformed average fold change (FC) for 311 pY sites identified by LC/MS-MS in cells treated with either 100 nM PTX or 30 nM VBL. Average FC of N = 3 biological replicates for each condition are normalized to the mean of 4 DMSO samples, log_2_-transformed, and organized by hierarchical clustering. (B) Volcano plot depicting −log_10_(p value) calculated using Student’s t test versus log_2_(average FC) for individual pY peptides. PTX (blue) and VBL (red) nodes are significant at p < 0.05 and ± 1.6FC. (C) String network of significantly downregulated proteins in PTX-treated cells, along with associated GO terms, and false discovery rate (FDR)-corrected p value. (D) String network of significantly up regulated proteins in VBL-treated cells, along with associated GO terms, and FDR-corrected p value. Unconnected nodes or nodes not involved in GO pathways are not depicted in (C) and (D). Line thickness indicates strength of data support from the String Consortium (Szklarczyk et al., 2015). Red nodes are associated with both insulin receptor signaling and EGF receptor signaling.

We used LC-MS/MS (Zhang et al., 2005) on U251 cell lysates treated with DMSO, 100 nM PTX, or 30 nM VBL, which revealed 311 pY sites differentially regulated between the two MTA-treated conditions (Figure 7A; Data S1). To classify our proteomic hits, we used gene ontology (GO) enrichment analysis (Gene Ontology Consortium, 2006), which identified several distinctions and similarities between the two drug treatments (Figure 7B; Data S1). Several RTK substrates for genes involved in motility were downregulated in PTX or both treatments. PTX significantly reduced ACTB (β-actin) pY levels (Figure 7C; Data S1). This particular pY site on ACTB was also reduced in VBL-treated cells, but not statistically significant (FC = −0.74; p = 0.069; Data S1). Phosphorylation of β-actin Tyr-53 is involved in protein-protein interactions required for polymerization and impairs filament nucleation and assembly *in vitro* and in *Dictyostelium* amoebae (Liu et al., 2006). Phosphoinositide-3-kinase (PI3K) subunit pY sites were also reduced in both treatment groups (PI3KR1/3 in PTX and PI3KR1 in VBL; Figure 7C; Data S1). PI3K subunit knockdown or depletion in cells affects migration and polarization (Cain and Ridley, 2009), so these emerge as potential regulators of protrusion dynamics involved in cell response to MTAs.

Our analysis revealed increased RTK signaling in cells treated with VBL corresponding to specific pathways (Figure 7D). This included epidermal growth factor receptor (EGFR) and insulin receptor signaling pathways. Notably, direct stimulation of EGFR causes a rapid increase in pMLC, followed by increased traction forces and retraction of protrusions within minutes (Schneider et al., 2009), a similar response to the one we observed following VBL treatments (Figure S3). Upregulated pY sites on ephrins (EPHA3 and EPHB4; Figure 7D; Data S1) were another particularly interesting finding, since this pathway controls patterning during nervous system development (Poliakov et al., 2004). Our observation of both convergent and divergent pY signaling changes warrants future study to determine the possible roles of these pathways in cellular responses to MTAs.

## DISCUSSION

Convergent effects of low-doses of MTAs (~1 nM) include kinetic stabilization and disruption of F-actin protrusion dynamics that impair stiffness-sensitive polarization and migration. Higher MTA doses (~10-100 nM) also divergently influence motor-clutch system mechanics: taxane site MTAs reduce traction force with little change to F-actin flow speed, while *vinca* site MTAs increase traction force and F-actin flow. In this study we tested a mechanical reinforcement model for microtubules as an alternative explanation for our results, and find that key experimental observations fail to match model predictions. These accumulated results support a signaling-based model for how microtubules regulate motor-clutch-based migration.

Divergent changes in RTK signaling pathways motivate directly testable hypotheses for how signaling-based mechanisms regulate glioma cell migration. First, as our results suggest that VBL stimulates the EGFR pathway, directly stimulating EGFR would result in similar contraction and loss of protrusions (Schneider et al., 2009). Our results show that similar rounding occurs following VBL treatment (Figure S3). Second, treating cells with an EGFR inhibitor concurrently with VBL could “override” MTA effects that relate to EGFR signaling, as was the case with inhibiting myosin II. Similar experiments and motor-clutch model analysis as described in this study could probe microtubule-based Rho GTPase signaling pathways that have long been associated with MTA effects on motility (Chang et al., 2008; Heck et al., 2012).

We conclude that the effects of MTAs on migration are consistent with increasing overall module number (by increasing module nucleation rate), which suppresses the establishment of cell polarity. Recent work by Zhang et al. (2014) modeled microtubule-based control of cell shape and concluded that microtubules enforce cell polarity by transporting inhibitory signals away from the leading edge. MTAs disrupt this link, allowing inhibitors to accumulate, which suppresses protrusion, and leads to directional switching when a new leading edge forms in another region of the cell. Although our conclusions are similar in a broad sense (e.g., dynamic microtubules reduce the protrusion nucleation rate, k_nuc,0_), the incorporation of motor-clutch dynamics in the CMS allowed us to test and reject alternative hypotheses that MTA effects on migration are solely due to motor and clutch numbers or direct mechanical interactions between actin and microtubules.

In our simulations, F-actin protrusion dynamics emerge as key parameters that mediate stiffness-sensitive migration. Increasing k_nuc,0_ or reducing v_actin,max_ produced polarity loss similar to that observed on soft or poorly adhesive substrates, where clutch slippage promotes faster module turnover and impairs polarization (Bangasser et al., 2017; Klank et al., 2017). MTAs cause a dramatic loss of EB1-decorated plus-ends (Figures 1A and S1A), suggesting that kinetic stabilization limits the delivery rate of factors required for protrusive activity (e.g., reduce v_actin,max_). The molecular players involved in this microtubule-actin cross talk could include MAPs or microtubule-based complexes that can directly template F-actin assembly from microtubule plus-ends (Henty-Ridilla et al., 2016). In hippocampal neurons, local microtubule dynamics are necessary for bursts of F-actin assembly that drive axon extension (Winans et al., 2016), lending support to this hypothesis. EB1 and dynein also regulate cell protrusion architectures that are required for efficient migration in 3D collagen gels (Jayatilaka et al., 2018). The relationship between EB1 and F-actin dynamics may be particularly relevant to GBM, since EB1 overexpression is a prognostic factor for sensitivity to *vinca* MTAs in pre-clinical mouse models (Berges et al., 2014).

Our results identify biophysical mechanisms for MTAs effects on glioma cell migration, which likely have broader impacts in other cancer types where MTAs are commonly used, such as carcinomas (Dumontet and Jordan, 2010). Our mechanistic findings likely also apply to emerging MTAs that limit invasion and improve survival of pre-clinical GBM mouse models (Bergès et al., 2016). Combination therapies targeting RTK signaling pathways linked to microtubule dynamics may provide new ways to treat MTA-resistant tumors. For example, inhibiting spleen tyrosine kinase in PTX-resistant ovarian cancer cells restores microtubule sensitivity (Yu et al., 2015). Outside oncology, MTAs show promise in tissue engineering applications, as evidenced by assembly promoters facilitating nerve regeneration following spinal cord injury (Ruschel et al., 2015). We suggest that biophysical models, such as the CMS, will help guide future clinical applications of MTAs (and other drugs) and systems-level analysis of cytoskeletal cross talk in migration.

## STAR*METHODS

## CONTACT FOR REAGENT AND RESOURCE SHARING

Further information and requests for resources and reagents should be directed to and will be fulfilled by the Lead Contact, David Odde (oddex002@umn.edu).

## EXPERIMENTAL MODEL AND SUBJECT DETAILS

### Cell Lines

Human U251 GBM cells (provided by Dr. G. Yancey Gillespie, University of Alabama-Birmingham) were authenticated in a previous publication (Bangasser et al., 2017). Cells were grown in DMEM/F12 (ThermoFisher, Cat#11320-033) containing 8% fetal bovine serum (ThermoFisher, Cat#10438), and 100 U mL^−1^ penicillin-streptomycin antibiotic (VWR Cat#45000-652) at 37°C with 5% CO_2_, and passaged in polystyrene flasks and tissue culture plates using phosphate buffered saline (PBS; VWR, Cat#21-031-CM) and 0.25% trypsin-EDTA (ThermoFisher, Cat#MT25053CI).

## METHOD DETAILS

### Pharmacological Agents

Vinblastine sulfate (Sigma) and paclitaxel (Sigma) were stored at 0.1-1 mM. Vincristine sulfate (Sigma) was stored at 10-100 μM and (−)-epothilone B (Sigma) was stored at 1-10 mM. Myosin II ATPase inhibitor (±)-blebbistatin (Tocris Bioscience) was stored at 50 mM. Phosphatase inhibitor Calyculin A (Santa Cruz Biotechnology) was stored at 10 μM. At the beginning of experiments, drug stocks were added to culture media at 2× working concentration and exchanged for half of the media volume in the dish. Added DMSO or drug volume did not exceed 0.5% of total solution volume in any condition, and the volume added was kept consistent between treatment groups.

### Transfection

U251 cells were transfected with pEB1-EGFP (a gift from Lynne Cassimeris, Lehigh University) (Piehl et al., 2004), pEGFP-α-tubulin; (Clontech), pEGFP-β-actin (a gift from Paul Letourneau, University of Minnesota) (Chan and Odde, 2008), or pmCherry-α-tubulin (a gift from Roger Tsien, University of California-San Diego) (Shaner et al., 2004) in antibiotic-free culture media using FuGENE® HD transfection reagent (Promega) according to manufacturer’s instructions at least 24 hours prior to plating.

### Semi-Automated Microtubule Tracking

No. 1.5 glass dishes (MatTek, Cat#P35G-1.5-14-C) were coated with 200 μg mL^−1^ rat-tail type I collagen (Corning) in PBS overnight and washed with PBS prior to plating cells. U251 cells expressing EB1-eGFP were imaged using the Nikon TiE microscope with a 100×/1.49 NATIRF lens and 1.5× zoom lens, LED illumination (SpectraX; Lumencor Inc., Beaverton, OR), and a Zyla 5.5 sCMOS camera (42 nm spatial sampling; Andor Technology, Belfast, UK). Streaming acquisition (300 ms per frame for 30 s) of regions of the cell periphery containing EB1 signal were obtained using a 470 nm LED, eGFP/mCherry filter set (Chroma, Cat#89021), and triggered acquisition mode in NIS Elements (Nikon). Individual microtubules were tracked using the TipTracker semi-automated plus-end localization algorithm (used without modification) (Prahl et al., 2014) with settings enabled for EB1 signal tracking (Seetapun et al., 2012). Microtubules were eligible for tracking if individual plus-ends could be unambiguously tracked (e.g., did not cross another microtubule) for at least 15 frames (4.5 s). Individual EB1 comet velocities were estimated as the mean of the frame-to-frame length change divided by the time between frames (Figures S1C and S1D).

### Tubulin FRAP

U251 cells expressing eGFP-α-tubulin were plated on type I collagen-coated 14 mm No. 1.5 glass dishes (MatTek), as described above. Cells were imaged on the Nikon TiE microscope using the 100×/1.49NA TIRF lens and Zyla 5.5 sCMOS camera (65 nm spatial sampling). A 100 mW 488 nm Argon ion laser was focused to a 1 μm diameter spot in the center of the camera field using at 50/50 beamsplitter and the Ti-PAU attachment on the microscope stand. Simultaneous streaming acquisition (100 ms per frame for 10 s) with an eGFP/mCherry filter set was provided by a 470 nm LED and triggered acquisition mode in NIS Elements. After 2 s of illumination (20 frames), the laser shutter was opened for 300 ms, resulting in bleaching of fluorescence signal within the laser spot to near background levels. A Uniblitz VS35 shutter and VMM-TI shutter driver provided control for laser shutter timing.

Fluorescence recovery was measured from time-lapse videos using a custom MATLAB script based on a previously published method (Castle et al., 2017). Average fluorescence signal was measured for each frame within the bleach region, and a separate, user-defined region distal to the bleach zone to correct for photobleaching that occurs during imaging. In each experiment, intensity values for both regions were normalized to the first 10 frames, and then a normalized, corrected intensity value (I_BC_) was calculated for all frames using Equation 2.

(Equation 2) $$I_{BC}=\frac{I_{bleach}}{I_{corr}}$$

I_bleach_ and I_corr_ are the normalized bleach and correction region intensities, respectively. Next, all frames post-bleach were fit to a single exponential function with two adjustable parameters.

(Equation 3) $$F_{recover}\left(t\right)=1-exp\left(-\lambda t\right)$$

In Equation 3, F_recover_(t) is the normalized fluorescence intensity at post-bleach time t, and λ is a constant that determines the timescale of recovery. Mobile fraction was calculated as the fitted quasi-steady state value from the last frame.

### Casting And Functionalizing PAGs

Polyacrylamide gel (PAG) substrates of varying Young’s modulus were prepared as described previously (Bangasser et al., 2017). Glass surfaces of 35 mm glass bottom dishes (MatTek, Cat#P35G-0-20-C) were etched with 0.1 N NaOH, followed by successive treatments with 3-aminopropyl-trimethoxysilane and 0.5% glutaraldehyde. Treated dishes were washed in deionized water (diH_2_O), allowed to air dry, and stored in a desiccator prior to use. Prepolymer solutions for PAG substrates were prepared by mixing acrylamide (40% by volume; Fisher Scientific, Hampton, NH), bis-acrylamide (2% by volume; Fisher), 1M HEPES (4-(2-hydroxyethyl) piperazine-1-ethanesulfonic acid, pH 8.5; Sigma), and diH_2_O in the appropriate ratios (see below), and degassing for 30 minutes. For traction force microscopy experiments, 0.2 μm crimson fluorescent microspheres (Invitrogen) were added to the prepolymer mix. Polymerization was initiated by adding 0.6% ammonium persulfate (10% w/v; Bio-Rad Laboratories, Hercules, CA) and 0.4% N,N,N,N-tetramethyl ethylenediamine (TEMED; BioRad Laboratories, Hercules, CA), and briefly mixing. A 4 μL bead of prepolymer was pipetted onto the treated glass surface and gently flattened under a clean 12 mm coverglass. Following polymerization, PAGs were rehydrated in 50 mM HEPES (pH 8.5) and the second cover glass removed with forceps.

PAGs were washed in several exchanges of 50mM HEPES before 250 μL of Sulfo-SANPAH (Sigma) in 50 mM HEPES was applied directly to the gel surface. PAGs were irradiated in 320-350 nm light for 8 minutes This entire process was repeated once, before incubating the PAG surface in a solution of 200 μg mL^−1^ rat tail type-I collagen (Corning) in PBS overnight at 4°C. The following day, dishes were washed in PBS, sterilized with UV light in a tissue culture hood for 15 minutes, and incubated in cell culture media prior to plating cells.

Young’s moduli were previously reported for the PAG recipes used in this study (Bangasser et al., 2017). The ratio of acrylamide/bis-acrylamide (base/cross-linker) and average Young’s modulus are: 3%/0.1% (0.74 kPa), 4%/0.2% (4.6 kPa), 5%/0.1% (9.3 kPa), 10%/0.1% (19.8 kPa), 20%/0.1% (98.5 kPa), and 20%/0.9% (195 kPa).

### Cell Shape and Random Motility

Phase contrast images were acquired using a 10×/0.25 NA Ph1 lens on a Nikon TiE epifluorescence microscope, with illumination provided by a white light LED (CoolLED Ltd., Andover, UK). Stage control was provided by a motorized stage (Prior Scientific, Cambridge, UK) and perfect focus system (Nikon) under control of NIS Elements Software (Nikon). For spread area, aspect ratio, or random motility coefficient measurements, cells were imaged every 15 minutes for 8–16 hours, starting at least 1 hour after addition of drugs or DMSO. For short-term spread area and aspect ratio experiments in Figure S3, cells were imaged every 3 minutes for 1 hour before and 1 hour immediately following addition of drugs or DMSO. Cell environments were maintained at 37°C and 5% CO_2_ using a BoldLine stagetop incubator (OkoLab Llc., Ottaviano, Italy).

Spread area, aspect ratio, and xy positions of individual cells were obtained from time-lapse videos using a custom MATLAB script, as previously described (Bangasser et al., 2017). Random motility coefficient fits were set to centroid positions using a random walk model (Equation 4).

(Equation 4) $$\mu =\frac{r^{2}\left(t\right)}{4t}$$

In Equation 4, μ is the random motility coefficient, t is the sampling time, and < r^2^ > is the mean-squared displacement. For each cell, Equation 4 was fit to MSD values for half of the individual cell tracking duration using the overlap method (Dickinson and Tranquillo, 1993). Cells were excluded when they entered mitosis, detached from the substrate, underwent apoptosis, or could not be unambiguously tracked as individual cells for at least 6 hours (24 frames).

### Traction Force Microscopy

TFM images were acquired using a Nikon TiE epifluorescence microscope with a Zyla 5.5 sCMOS camera (Andor) and a 40×/0.95NA Ph2 lens with 1.5× intermediate zoom (60× total magnification, 108 nm spatial sampling). Cells were maintained at 37°C and 5% CO_2_ for the duration of imaging. At each stage position, a phase contrast image of the cell body was acquired. Next, an image of fluorescent beads at the top surface of the gel was captured using a 575/25 nm LED and eGFP/mCherry filter set with LED fluorescence illumination from a SpectraX Light Engine (Lumencor). Media in dishes was carefully removed, cells were detached with 0.25% trypsin/EDTA (Corning), and fluorescence images of beads in the absence of cell-induced deformation were acquired at saved stage positions.

Sub-pixel bead position measurements were estimated using a previously described method (Bangasser et al., 2017). Briefly, a morphological top hat filter removes noise from the pre- and post-trypsin bead images. The cell body outline was identified from the phase contrast image using a Sobel filter, and used as a mask to exclude beads that may be moved by the cell from registration. Post-trypsin images were aligned to the pre-trypsin image, and bead displacements between the strained and relaxed images were computed using particle image velocimetry (PIV). Images were binned into 48-pixel (5.28 μm) square windows and 2D cross-correlation was performed between the two images. A fine displacement field was created with a 24-pixel (2.64 μm) square window, using the cross-correlation maximum as the initial guess. Fine windows were overlapped for a final lattice spacing of 12 pixels (1.32 μm). Stress and displacement vectors were obtained by solving the inverse Boussinesq problem in Fourier space (Butler et al., 2002). Integrating the product of the stress and displacement vectors over the entire image gives substrate strain energy, in pico-Joules (pJ), as in Equation 5.

(Equation 5) $$U_{strain}=\int \int \vec{T}\left(\vec{r}\right)\cdot \vec{u}\left(\vec{r}\right)\;dxdy$$

### F-actin Flow Measurements

U251 cells were transfected with eGFP-β-actin or co-transfected with mCherry-α-tubulin before plating on PAGs or 14 mm No. 1.5 glass dishes (MatTek) that had been coated with 200 μg mL^−1^ rat-tail type I collagen (Corning) in PBS. Images were acquired every 2 s for 2 minutes using a Nikon TE200 epifluorescence microscope equipped with a CoolSnap HQ^2^ CCD camera (Photometrics, Tucson, AZ), BioPrecision motorized stage (Ludl Electronic Products Inc., Hawthorne, NY), and Plan Apo 60×/1.4 NA Ph3 immersion oil lens (108 nm spatial sampling). Fluorescence illumination was provided by a PhotoFluorII® metal halide light source (89 North) and single-band eGFP filter set (Chroma, Cat#49020). The system was controlled using Meta Morph v7.0.2 (Molecular Devices). Cells were maintained at 37°C and 5% CO_2_ for the duration of imaging using a stagetop incubation system (OkoLab). Kymographs were generated from actin features at the cell periphery using Fiji; typically 2-5 regions were obtained per cell. Individual F-actin flow velocities were calculated from kymographs by spatial cross-correlation using a previously described MATLAB script (Chan and Odde, 2008). F-actin flow velocities are reportedly similar in U251 cells expressing EGFP-β-actin and in un-transfected cells using time-lapse phase contrast microscopy (Bangasser et al., 2017), suggesting that expression of fluorescently labeled actin does not affect flow speed.

Co-transfected cells were imaged every second for 1 minute using the previously described Nikon TiE microscope system with 470 nm and 565 nm LED lines, an eGFP/mCherry filter set, and triggered acquisition mode in NIS Elements. A 100×/1.49 NA TIRF lens (Nikon) yielded 65 nm spatial sampling. F-actin flow speeds were otherwise analyzed from kymographs as described above. In co-transfected cells, F-actin flow measurement regions were labeled and scored by the user as containing a microtubule (+MT) or not (−MT). An independent user that was blinded to the results of initial analysis scored regions and obtained similar results.

### Motor-Clutch Model Simulations

In a motor-clutch model for cell traction (Bangasser et al., 2013; Chan and Odde, 2008), simulated cells contain an ensemble of myosin II motors (n_motor_), each capable of generating a stall force, F_motor_ that slide an F-actin bundle at velocity v_motor_ in the absence of opposing load. A set of molecular clutches (n_clutch_) are modeled as linear elastic springs with characteristic stiffness κ_clutch_ and connected to a compliant substrate spring of stiffness κ_substrate_. Individual clutches bind the F-actin bundle at a rate k_on_ and transmit force to the compliant substrate as they are extended by retrograde flow. As force builds on the substrate, retrograde flow slows from its unloaded rate with a characteristic force-velocity relationship (Chan and Odde, 2008). Force on bound clutches increases their unbinding rate from its base level (k_off_) as a slip bond with a characteristic bond scaling force (F_bond_). For additional details on simulation parameters for the reference condition, see Methods S1. We simulated the effects of pharmacological inhibitors by scaling the following parameters by the following multipliers: paclitaxel scales n_motor_ and n_clutch_ by 0.6×; vinblastine scales n_motor_ by 1.5× and n_clutch_ by 1.2×; calyculin A scales n_motor_ by 3×; and blebbistatin scales n_motor_ by 0.25×. All other changes from the reference numbers for other parameter sets are given in the figure panels or figure legends.

We used the direct implementation of a Stochastsic Simulation Algorithm (SSA) to guide simulation progression (Gillespie, 1977), as this only requires two random numbers to be generated at each time step, regardless of the number of events. Based on the current state of the system, the individual rates for clutch binding and unbinding (k_i_) are summed, and an event time generated from the summed rates, according to Equation 6.

(Equation 6) $$\Delta t=\frac{-\ln\left(URN_{1}\right)}{\sum_{i=1}^{n_{clutch}}k_{i}}$$

URN_1_ is a random number from the uniform distribution. Next, a second random number URN_2_ is drawn and an event selected subject to the conditions set in Equation 7.

(Equation 7) $$\sum_{i=1}^{j-1}k_{i}<URN_{2}<\sum_{i=1}^{j}k_{i}<\sum_{i=1}^{j+1}k_{i}$$

This condition maintains proportionality between individual rates, so that events with a higher rate constant are more likely to be selected. All simulations were allowed to proceed for 10^6^ events.

### Simulation of Microtubule Mechanics

To simulate mechanically linked microtubules and F-actin, we added a second linear elastic spring with characteristic stiffness (κ_MT_), estimated using values from published literature (see Estimating Simulated Microtubule Spring Constant). We assumed that spectraplakins or other proteins that are capable of binding both actin and tubulin mediate connectivity between the filament types (Suozzi et al., 2012; Wu et al., 2008). These were included as a set of cross-linkers (n_x-link_) that are attached to the compliant microtubule spring and transiently bind F-actin with slip bond unbinding behavior. We assume that cross-linker spring stiffness (κ_x-link_) and characteristic cross-linker bond force (F_bond,x-link_) are the same as clutches. Cross-linkers bind and unbind in a similar fashion to clutches, engaging the microtubule spring with a pseudo-first-order binding rate and a single exponential force-dependent unbinding rate (k_off,x-link_) (Chan and Odde, 2008). We assumed a pseudo-first order binding rate for cross-linkers to actin (k_on,x-link_), because plus-end tracking MAPs may locally concentrate to micromolar concentrations (Seetapun et al., 2012) and F-actin is concentrated at the cell periphery, where traction forces are transmitted.

Force is transmitted to both the compliant substrate (via clutches) and the compliant microtubule (via cross-linkers). The two forces are summed to oppose the myosin II motors that power F-actin flow. Actin velocity (v_flow_) thus depends on both the microtubule compression force and substrate traction force.

(Equation 8) $$v_{flow}=v_{motor}\left(1-\frac{\kappa_{MT}x_{MT}+\kappa_{sub}x_{sub}}{F_{motor}n_{motor}}\right)$$

In Equation 8, v_motor_ is the unloaded myosin II velocity, κ_sub_ and x_sub_ are the substrate spring constant and displacement, respectively, and κ_MT_ and x_MT_ are the microtubule spring constant and compression, respectively. At each simulation step, substrate position is solved analytically as a force balance equation between engaged clutches and the substrate.

(Equation 9) $$x_{sub}=\frac{\kappa_{clutch}\sum_{j=1}^{n_{engaged}}x_{clutch,j}}{\kappa_{sub}+n_{engaged}\kappa_{clutch}}$$

A similar force balance to Equation 9 is solved between cross-linkers and the microtubule.

(Equation 10) $$x_{MT}=\frac{\kappa_{x-lin\kappa }\sum_{j=1}^{n_{engaged}}x_{x-link,j}}{\kappa_{MT}+n_{engaged}\kappa_{clutch}}$$

In Equation 10, n_engaged_ cross-linkers are bound to the compliant microtubule, and κ_x-link_ is the stiffness of an individual cross-linker. Simulations of the microtubule-motor-clutch model were otherwise run as described previously (Bangasser et al., 2013), but with additional possible events for binding and unbinding of microtubule-actin cross-linkers at each time step.

### Estimating Simulated Microtubule Spring Constant

Microtubule flexural rigidity (EI_MT_) has been measured using a number of *in vitro* techniques and tubulin preparations (e.g., PTX-stabilized, co-precipitated with or without MAPs), which were reviewed in VanBuren et al. (2005). This list was updated to include more recent data from Hawkins et al. (2013). We excluded values for purified tubulin alone and collected an aggregated average value from studies measuring EI_MT_ in microtubules co-precipitated with MAPs. EI_MT_ values for tubulin with MAPs ranged from 16-43 (×10^−24^ Nm^2^). Some studies also measured EI_MT_ for PTX-stabilized microtubules, which have slightly lower EI_MT_ than values measured from dynamic tubulin, or microtubules stabilized by other methods. A previous study reports that there is no synergistic or antagonistic effect of adding PTX to microtubules assembled in the presence of MAPs (Hawkins et al., 2013), so we did not consider a separate value for EI_MT_ in MTA-treated cells.

Equation 11 relates flexural rigidity to an elastic (Young’s) modulus, E.

(Equation 11) $$E=\frac{EI_{MT}}{I_{MT}}$$

I_MT_ is the second moment of area for the microtubule. Modeling the microtubule as a hollow cylinder with an internal diameter of 12 nm and external diameter of 25 nm, the second moment along the cylinder axis (I_MT,z_) is 5.8×10^−31^ m^4^. Using the average flexural rigidity (EI_MT_ = 21.9×10^−24^ N m^2^), we obtain a Young’s modulus of 3.8×10^7^ N m^−2^ for the microtubule.

We model the microtubule as undergoing axial compressive loading from F-actin flow along its longitudinal axis (Brangwynne et al., 2006; Soheilypour et al., 2015). The distal end of the microtubule is considered a fixed point (e.g., anchored at the centrosome). Microtubule buckling and relaxation occurs at the seconds timescale or faster (Bicek et al., 2009), so we ignore viscous relaxation and assume that the microtubule behaves as a purely elastic material. Equation 12 relates Young’s modulus (E) to a deformation, F is an applied load, A is the cross-sectional area, ΔL is the length change due to deformation, and L_0_ is the rest length

(Equation 12) $$E=\frac{FL_{0}}{A\Delta L}$$

Rearranging this expression yields Equation 13, which contains an elastic spring constant term (κ), given in Equation 14.

(Equation 13) $$F=\left(\frac{EA}{L_{0}}\right)\Delta L$$

(Equation 14) $$\kappa =\frac{EA}{L_{0}}$$

The cross-sectional area of a microtubule with a 25 nm outer diameter and 12 nm inner diameter is 3.8×10^−16^ m^2^. Fora 10 μm long microtubule, Equation 14 yields a spring constant of κ_MT_ = 1.4 pN nm^−1^. Microtubules that track along actin filaments or the cell cortex distal to the tip may experience compressive loading from locations distal to the tip, which changes the effective length, and thus the spring constant. We used κ_MT_ = 1 pN nm^−1^ as a base value for simulations (see Methods S1 for other simulation parameter values).

### Stochastic Cell Migration Simulator

Simulated cell migration in a compliant microenvironment using a motor-clutch model is described previously (Bangasser et al., 2017; Klank et al., 2017). Briefly, the simulated cell consists of modules that represent cell protrusions attached to a central cell body by elastic springs (spring constant κ_cell_) representing the simulated cell’s nucleo-cytoskeletal compliance. Each module consists of a functioning copy of the motor-clutch model (Chan and Odde, 2008). The distal end of each module connects to the compliant substrate (substrate position given by x_sub,j_) at a reference point (x_ref,j_), where j is the module index variable. An ensemble of clutches associated with the cell body (n_clutch,cell_) generate traction forces as the cell body moves, but are not subject to direct motor forces, as is the case for clutches in modules.

Modules extend via actin polymerization at a rate v_actin_, which depends on the maximal polymerization velocity (v_actin,max_) and the ratio of G-actin (A_G_) to total actin (A_total_), as described by Equation 15.

(Equation 15) $$v_{actin}=v_{actin,max}\left(\frac{A_{G}}{A_{total}}\right)$$

Module nucleation (at rate k_nuc_) similarly depends on the ratio of A_G_ to A_tot_, which scales the maximal nucleation rate (k_nuc,0_) by Equation 16.

(Equation 16) $$k_{nuc}=k_{nuc,0}{\left(\frac{A_{G}}{A_{total}}\right)}^{4}$$

A_total_ represents the total possible length of the cell, and constrains the F-actin lengths for each module (A_F,j_) for a cell with n_mod_ modules, by the relationship in Equation 17.

(Equation 17) $$A_{total}=A_{G}+\sum_{j=1}^{n_{mod}}A_{F,j}$$

Material balances constrain the total number of motors (n_motor_) and clutches (n_clutch_) and F-actin length assigned to new modules, and these components are allocated to new modules as previously described (Bangasser et al., 2017). Capping of a single module occurs with a first-order rate constant (k_cap_) and stochastically terminates actin polymerization. Modules are destroyed and their contents (e.g., F-actin, motors, clutches) returned to the cell pool if they pass a minimum threshold length (L_min_). Cell position (x_cell_) is calculated after each event by iteratively solving a force balance between traction forces generated by modules and the cell body, as previously described (Bangasser et al., 2017; Klank et al., 2017).

Simulation architecture and initial conditions are as previously described (Bangasser et al., 2017). For additional details on simulation parameters for the reference condition, see Methods S1. All changes for different parameter sets are given in figure panels or figure legends. An SSA, as described in Equations 6 and 7 and Gillespie (1977) governed event selection (e.g., binding or unbinding of clutches, module capping, or module nucleation) and simulation progression. The vehicle control parameter set used as a reference condition (white circles in Figures 6, S5, and S6) corresponds to the lower number of motors and clutches in Bangasser et al. (2017). Individual runs progressed for 5-8 hours of simulated time, discarding the first hour before the simulation reaches steady state. Equation 4 was used to calculate random motility coefficients for each individual cell from cell position at 15-minute sampling intervals. A polygon shape was fit to the module and cell body positions, and used to estimate aspect ratio (Bangasser et al., 2017). Total traction force was calculated as the summed substrate forces on all modules and the cell body. F-actin flow was averaged over all modules at each time point.

### Generation and Preparation of Cell Lysates

U251 cells were plated on 10 cm tissue culture dishes at a density of 10^6^ cells per dish. The following day, culture media was exchanged for media containing DMSO, 100 nM paclitaxel, or 30 nM vinblastine for 1 hour. Cells were washed with PBS and lysed on ice in a solution containing 8 M urea and 1 mM sodium orthovanadate. Lysates were collected using a cell scraper, transferred to 15 mL centrifuge tubes (Corning), and stored at −80°C. Protein concentrations were verified using a Pierce bicinchoninic acid (BCA) protein assay kit (ThermoFisher). Three dishes were pooled to generate a single sample for each condition.

Cell lysates were reduced with 10 mM dithiothreitol (DTT)for 1 hour at 56°C, alkylated with 55 mM iodoacetamide (IAA)for 1 hour at room temperature, and diluted 8-fold with 100 mM ammonium acetate (pH 8.9). Proteins were digested with 1 μg sequencing grade trypsin (Promega) per 50 μg protein, and rotated overnight at room temperature. Enzymatic activity was quenched with glacial acetic acid, and peptides were desalted and concentrated using C18 Sep-Pak cartridges (Waters). Peptides were eluted from cartridges using 40% acetonitrile in 0.1% acetic acid. Organic solvents were evaporated with a vacuum centrifuge. Aliquots of 400 μg per sample were frozen in liquid nitrogen and subsequently lyophilized.

Lyophilized peptides were labeled using tandem mass tag (TMT) 10-plex Mass Tag Labeling Kits (Thermo Fisher). Three biological replicates of each condition (DMSO, VBL, PTX) along with a DMSO technical replicate were labeled by resuspending peptides in 70 μL ethanol and 30 μL of 0.5 M triethylammoniumbicarbonate and incubating with TMT (resuspended in 30 μL anhydrous acetonitrile) for 1 hour at room temperature. Samples were then combined and dried down using a vacuum centrifuge. Phosphotyrosine (pY) enrichment of labeled samples was performed by phospohotyrosine immunoprecipitation (IP) followed by immobilized metal affinity chromatography (IMAC) (Emdal et al., 2017). Resuspended peptides were incubated with protein G agarose beads conjugated with 12 μg 4G10 (Millipore) and 9 μg PT-66 (Sigma) overnight at 4°C. Phospho-tyrosine peptides were eluted for 30 minutes with 100 mM glycine (pH 2.5).

IMAC enrichment was performed using a column made in house column (200 μm ID × 10 cm) packed with Poros 20 MC beads (Thermo Fisher). IMAC columns were washed with EDTA and milli-Q, each for 5 minutes, followed by 100 mM Fe(III)-chloride (FeCl_3_) for 10 minutes, and then 0.1% acetic acid for 5 minutes at 10-12 μL min^−1^. IP elution was loaded onto the IMAC column at a flow rate of 1 μL min^−1^, then rinsed (100 mM sodium chloride, 25% acetonitrile in 0.1% acetic acid)] for 10 minutes to remove non-phosphorylated peptides. Following the rinse step, 0.1% acetic acid was washed over the column for 5 minutes at 10-12 μL min^−1^. Finally, retained peptides were eluted with 250 mM NaH_2_PO_4_ (pH 8.0) onto a precolumn (100 μm ID × 10 cm) packed with 10 μm C18 beads (YMC gel, ODS-A, 12 nm, S-10 μm, AA12S11) connected in series to the IMAC column. The precolumn was then washed with 0.1% acetic acid, and connected in series to an analytical capillary column prepared in house (50 μm ID × 12 cm) and packed with 5 μm C18 beads (YMC gel, ODS-AQ, 12 nm, S-5 μm, AQ12S05) with an integrated electrospray tip (~1 μm orifice).

### Liquid Chromatography-Mass Spectrometry

Phosphopeptides were eluted using a 140 minute gradient ranging from 9% to 70% acetonitrile in 0.2 M acetic acid at a flow rate of 0.2 mL min^−1^. The flow split was approximately 10,000:1, with a final electrospray flow rate of ~20 nL min^−1^. Phosphopeptides were analyzed using a Q Exactive HF-X Hybrid Quadrupole-Orbitrap mass spectrometer (Thermo Fisher). Standard mass spectrometric parameters were as follows: spray voltage, 2.5 kV; no sheath or auxiliary gas flow, heated capillary temperature, 250°C; S-lens radio frequency level of 40%. The HF-X was operated in data-dependent acquisition mode. Full-scan mass spectrometry spectra [mass/charge ratio (m/z), 300 to 2,000; resolution, 60,000] were detected in the Orbitrap analyzer after accumulation of ions at 3e6 target value based on predictive AGC from the previous scan. For every full scan, the 15 most intense ions were isolated (isolation width of 0.4 m/z) and fragmented (collision energy (CE): 33%) by higher energy collisional dissociation (HCD) with a maximum injection time of 350 ms and 30,000 resolution. Dynamic exclusion was set to 15 s.

To correct for variation in sample amount in each TMT channel, approximately 50 ng of the IP supernatant was loaded onto an acidified precolumn, attached in series to an analytical column, and analyzed by LC-MS/MS on an LTQ Orbitrap XL mass spectrometer as previously described (Emdal et al., 2017). Peptides were eluted using a 90 minute gradient, ranging from 9% to 70% acetonitrile in 0.2 M acetic acid at a flow rate of 0.2 mL min^−1^. Raw mass spectral data files were analyzed using Proteome Discoverer version 1.4.1.14 (DBversion: 79; Thermo Fisher) and searched against the human SwissProt database (20,194 sequence entries) using Mascot version 2.4 (Matrix Science). TMT reporter quantification was extracted using Proteome Discoverer. Spectra were matched using an initial mass tolerance of 10 ppm on precursor masses and 15 mmu for fragment ions. The search included fixed modifications of cysteine carbamidomethylation, TMT-labeled lysine and TMT-labeled protein N-terminals. Variable modifications were oxidized methionine and phosphorylation of serine, threonine, and tyrosine. Minimal peptide length was seven amino acids. Peptides containing a phosphorylated tyrosine residue were manually validated according to previously described criteria using Computer Aided Manual Validation (CAMV) as previously described (Curran et al., 2013; Nichols and White, 2009). TMT reporter ions were added together for identical and miscleaved peptides, and normalized using the median TMT quantification value for all peptides with ion score > 25 from the supernatant peptide analysis. For each phosphopeptide, relative quantification was calculated as a log_2_-transformed Fold Change for each sample from the average TMT value for DMSO control.

## QUANTIFICATION AND STATISTICAL ANALYSIS

Measurements represent pooled data from at least two biological replicates. Number of cells in a particular experimental condition is given in figure legends. Similarly, numbers of CMS runs for a particular parameter set are given in figure legends. Error bars on graphs represent mean ± SEM. In most cases SEM was calculated using number of cells. For microtubule plus-end tracking and F-actin flow speed, SEM was calculated using the number of measurements, since variance between individual measurements was larger than variance between cell average values. No prior estimation of sample sizes was conducted. No data points were excluded from the study.

Pairwise comparisons between groups were made using a 1-way ANOVA or Kruskal-Wallis test, unless another test is stated in the figure legends. The non-parametric Kruskal-Wallis test was chosen in most cases because it makes no assumptions about the shape of the underlying distribution and allows for comparison of groups of varying size. Dunn-Sidăk multiple comparisons correction was used when comparisons included more than two groups. For calculating average trends in strain energy as a function of MTA concentration (Figure S2), individual data points were fit to a first-order linear function (y = ax + b, where y represents strain energy and x represents MTA concentration). Slope p values were obtained using the F-test statistic against the null hypothesis that strain energy is unaffected by MTA (a = 0). To assess changes in motility using the CMS, we performed pairwise Kruskal-Wallis tests between the test condition and reference parameters at each κ_sub_ value. We defined loss of stiffness sensitive motility if a test parameter set produced two or more significant reductions in motility for substrate spring stiffness values between κ_sub_ = 1 pN nm^−1^ and κ_sub_ = 100 pN nm^−1^, where motility values are largest in the reference condition. All statistical analysis was performed using the MATLAB Statistics Toolbox, and statistical significance was considered if p < 0.05.

To identify significant changes between groups in the phosphoproteomics data, a Student’s t test was performed for each peptide, and peptides with p < 0.05 were considered significant. Fold-change cutoffs were applied at greater than 1.6-fold change for upregulated peptides and less than 0.625-fold change for downregulated peptides. Significantly upregulated or downregulated peptides were queried against the STRING database (v10.5) to identify interconnected protein networks (Szklarczyk et al., 2015). Gene ontology (GO) enrichment analysis was performed on the same peptide groups for terms related to biological processes and cellular components (Gene Ontology Consortium, 2006). Significant term enrichment was determined at p < 0.05 by Fisher exact test with false discovery rate (FDR) correction.

## DATA AND SOFTWARE AVAILABILITY

All experimental data, analysis scripts, and simulation codes used in this study are available upon request from the corresponding author. MATLAB scripts for the motor-clutch model and CMS are also freely available on the Odde laboratory website (http://oddelab.umn.edu) and the University of Minnesota Physical Sciences-Oncology Center website (http://psoc.umn.edu). Mass spectrometry proteomics data are deposited to the ProteomeXchange Consortium via the PRIDE partner repository (Vizcaíno et al., 2016) with the dataset identifiers PRIDE: PXD008939 and 10.6019/PXD008939. Dataset identifiers for TMT normalization data are PRIDE: PXD008940 and 10.6019/PXD008940.

## Supplementary Material

Data S1

Document S1

Video S1

Video S2

Video S3

Video S4

## Figures and Tables

**Table:** KEY RESOURCES TABLE

REAGENT or RESOURCE	SOURCE	IDENTIFIER
Chemicals, Peptides, and Recombinant Proteins		
FuGENE HD transfection reagent	Promega	Cat# E2311
Dimethyl sulfoxide	Millipore Sigma	Cat# D2650; CAS:67-68-5
Paclitaxel, from *T. brevifolia*	Millipore Sigma	Cat# T7402; CAS:33069-62-4
Vinblastine sulfate	Millipore Sigma	Cat#V1377; CAS:143-67-9
Vincristine sulfate	Millipore Sigma	Cat#V8879; CAS:2068-78-2
(−)-epothilone B	Millipore Sigma	Cat#E2656; CAS:152044-54-7
(±)-blebbistatin	Tocris Bioscience, Bristol, UK	Cat#1760; CAS:674289-55-5
Calyculin A	SantaCruz Biotechnology	Cat#sc24000; CAS:101932-71-2
Collagen I, rat	ThermoFisher Scientific	Cat#CB40236
Acrylamide (40% w/v)	ThermoFisher Scientific	Cat#BP1402
Bis-acrylamide (2% w/v)	ThermoFIsher Scientific	Cat#BP1404
(3-aminopropyl)trimethoxysilane	Sigma Aldrich	Cat#281778; CAS:13822-56-5
Glutaraldehyde, grade I	Millipore Sigma	Cat#G7776; CAS:111-30-8
HEPES	Millipore Sigma	Cat#H4034; CAS:7365-45-9
Ammonium persulfate	Bio-Rad Laboratories	Cat#1610700; CAS:7727-54-0
TEMED	Millipore Sigma	Cat#T9281; CAS:110-18-9
Sulfo-SANPAH	ThermoFisher Scientific	Cat#22589
0.2 μm diameter crimson fluorescent beads	Invitrogen	Cat#F8806
Sequencing grade modified trypsin	Promega	Cat#V5113
Critical Commercial Assays		
Pierce BCA Protein Assay	ThermoFisher Scientific	Cat#23225
Sep-Pak Plus C18 cartridge	Waters Corp., Milford, MA	Cat#WAT020515
TMT 10-plex isobaric label reagent set	ThermoFisher Scientific	Cat#90406z
Deposited Data		
Mass spectrometry proteomics data from MTA-treated U251 cells	This paper; Proteome XChange	Data S1; https://www.ebi.ac.uk/pride/archive
TMT normalization data	This paper; Proteome XChange	https://www.ebi.ac.uk/pride/archive
Experimental Models: Cell Lines		
Human: U251 GBM cell line	Dr. G. Yancey Gillespie, University of Alabama-Birmingham	N/A
Recombinant DNA		
Plasmid: pEB1-eGFP	Dr. Lynne Cassimeris, Lehigh University; Piehl et al., 2004	N/A
Plasmid: pEGFP-α-tubulin	Clontech	Cat#632349
Plasmid: pEGFP-β-actin	Dr. Paul Letourneau, University of Minnesota	N/A
Plasmid: pmCherry-α-tubulin	Dr. Roger Tsien, University of California-San Diego; Shaner et al., 2004	N/A
Software and Algorithms		
MATLAB	The MathWorks Inc.	https://www.mathworks.com/products/matlab.html
ImageJ	NIH	https://imagej.nih.gov/ij/
Origin v9.1 graphing and statistics software	OriginLab Corp., Northampton, MA	https://www.originlab.com
TipTracker_v3 MATLAB code	Prahl et al., 2014	http://oddelab.umn.edu
PolymerFRAP MATLAB code	This study; Castle et al., 2017	http://oddelab.umn.edu
CellTracker MATLAB code	Bangasser et al., 2017	http://oddelab.umn.edu
Traction Strain Energy MATLAB code	Bangasser et al., 2017	http://oddelab.umn.edu
FlowTrack MATLAB code	Chan and Odde, 2008	http://oddelab.umn.edu
Motor-Clutch Model MATLAB codes	This study; Chan and Odde, 2008	http://oddelab.umn.edu; http://psoc.umn.edu
Cell Migration Simulator v1.0 MATLAB code	Bangasser et al., 2017	http://oddelab.umn.edu; http://psoc.umn.edu
Proteome Discoverer v1.4	ThermoFisher Scientific	http://planetorbitrap.com/proteome-discoverer/#.W9j-AGhKg2w
Mascot v2.4	Matrix Science	http://www.matrixscience.com/
Other		
100 mW 488 nm Argon ion laser	Spectra-Physics, Santa Clara, CA	N/A
Uniblitz V35 shutter	Vincent Associates, Rochester, NY	N/A
VMM-TI shutter driver	Vincent Associates, Rochester, NY	N/A
Q Exactive HF-X Hybrid Quadropole-Orbitrap mass spectrometer	ThermoFisher Scientific	Cat#0726042
LTQ Orbitrap XL mass spectrometer	ThermoFisher Scientific	Cat#IQLAAEGAAPFADBMAOK

## Highlights

- Microtubule-targeting agents (MTAs) suppress mechanosensitive cell migration
- MTAs influence traction forces, and F-actin flows via a motor-clutch system
- Migration simulations predict that MTAs influence actin assembly or nucleation
- Mass spectrometry proteomics suggest MTA-specific signaling pathway changes
