# Supplementary material for: Microtubule-Based Control of Motor-Clutch System Mechanics in Glioma Cell Migration
Source: Cell Rep. Author manuscript; Available in PMC 2019 Jan 24. (PMC6345402; doi:10.1016/j.celrep.2018.10.101)
Supplement: Document S1 [file NIHMS1516185-supplement-Document_S1.pdf]

Cell Reports, Volume *25*

Supplemental Information

Microtubule-Based Control of Motor-Clutch System Mechanics in Glioma Cell Migration

Louis S. Prahl, Patrick F. Bangasser, Lauren E. Stopfer, Mahya Hemmat, Forest M. White, Steven S. Rosenfeld, and David J. Odde

Supplemental figures and legends

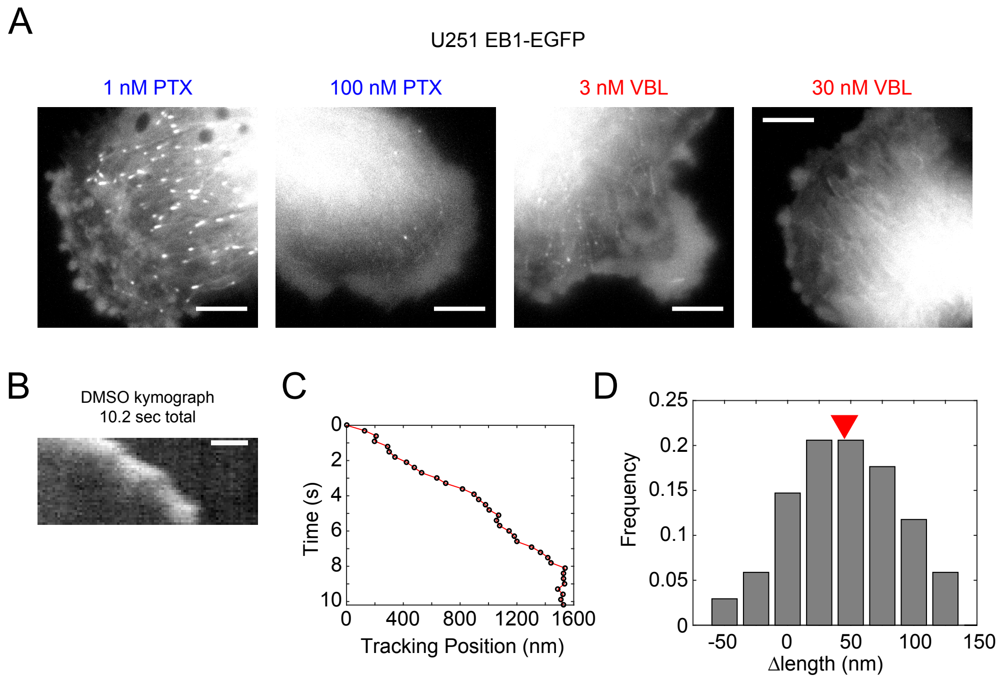

Figure S1. EB1-eGFP distribution in MTA-treated U251 cells and validation of growth velocity measurements from EB1 tracking. Related to **Figure 1** and **STAR Methods**.

(A) Images of EB1-eGFP distribution in U251 cells following the addition of MTAs at various doses. Loss of EB1-eGFP-decorated plus ends is observed in a dose-dependent fashion. Scale bars, 5 μm.

(B) Kymograph of EB1-eGFP signal at the end of a growing microtubule, recorded over a 10.2 second tracking interval. Scale bar, 500 nm.

(C) Microtubule tip position versus time for the kymograph in panel B, obtained using TipTracker_v3 (Prahl et al., 2014). Initial tracking position is set to x = 0.

(D) Histogram of incremental length changes for the length-time data in panel C. Red arrow shows the mean of the distribution (44 nm), which corresponds to a growth velocity of 147 nm s^{-1} for a 0.3 s acquisition interval.

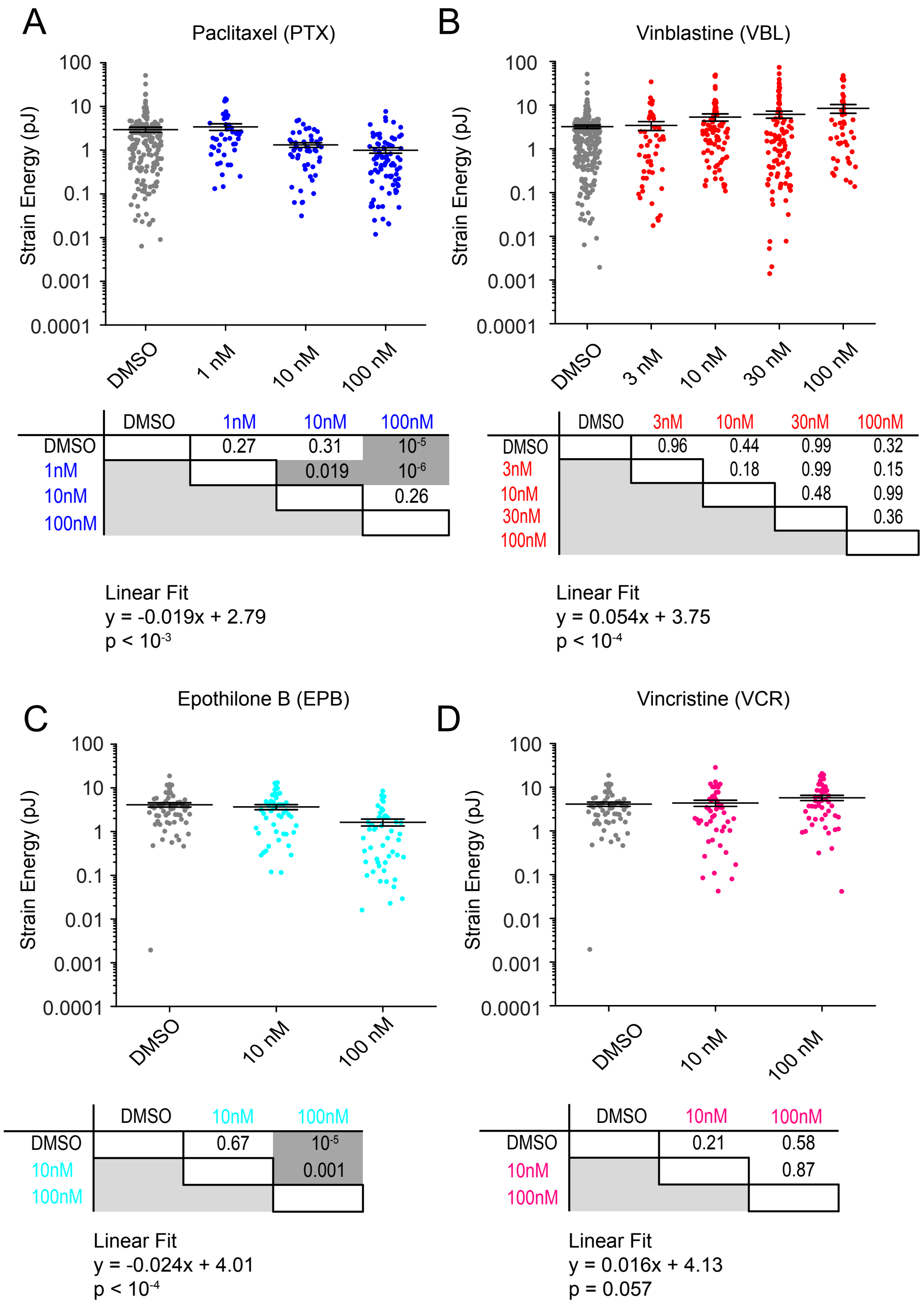

Figure S2. Traction strain energy trends for varying MTA dose and binding site. Related to **Figure 3**.

(A) Strain energy measurements for U251 cells on 9.3 kPa PAGs and treated with DMSO, 1 nM, 10 nM, or 100 nM PTX, n = 184, 44, 50, 84 cells. DMSO and 100 nM PTX groups are replicated from the 9.3 kPa condition in **Figure 3D**.

(B) Strain energy measurements for U251 cells on 9.3 kPa PAGs and treated with DMSO, 3 nM, 10 nM, 30 nM, or 100 nM VBL, n = 243, 52, 96, 100, 46 cells. DMSO and 30 nM VBL groups include data from the 9.3 kPa condition in **Figure 3D**.
(C) Strain energy measurements for U251 cells on 9.3 kPa PAGs and treated with DMSO, 10 nM, or 100 nM EPB, n = 59, 48, 50 cells.
(D) Strain energy measurements for U251 cells on 9.3 kPa PAGs and treated with DMSO, 10 nM, or 100 nM VCR, n = 59, 51, 46 cells. DMSO controls are repeated from panel C. Error bars represent mean ± SEM, pairwise statistics calculated by Kruskal-Wallis test with Dunn-Sidăk test. Non-weighted linear model (y = ax+b) was fit to all data points, and p-values compared to the null hypothesis (a = 0) were calculated using the F-test statistic.

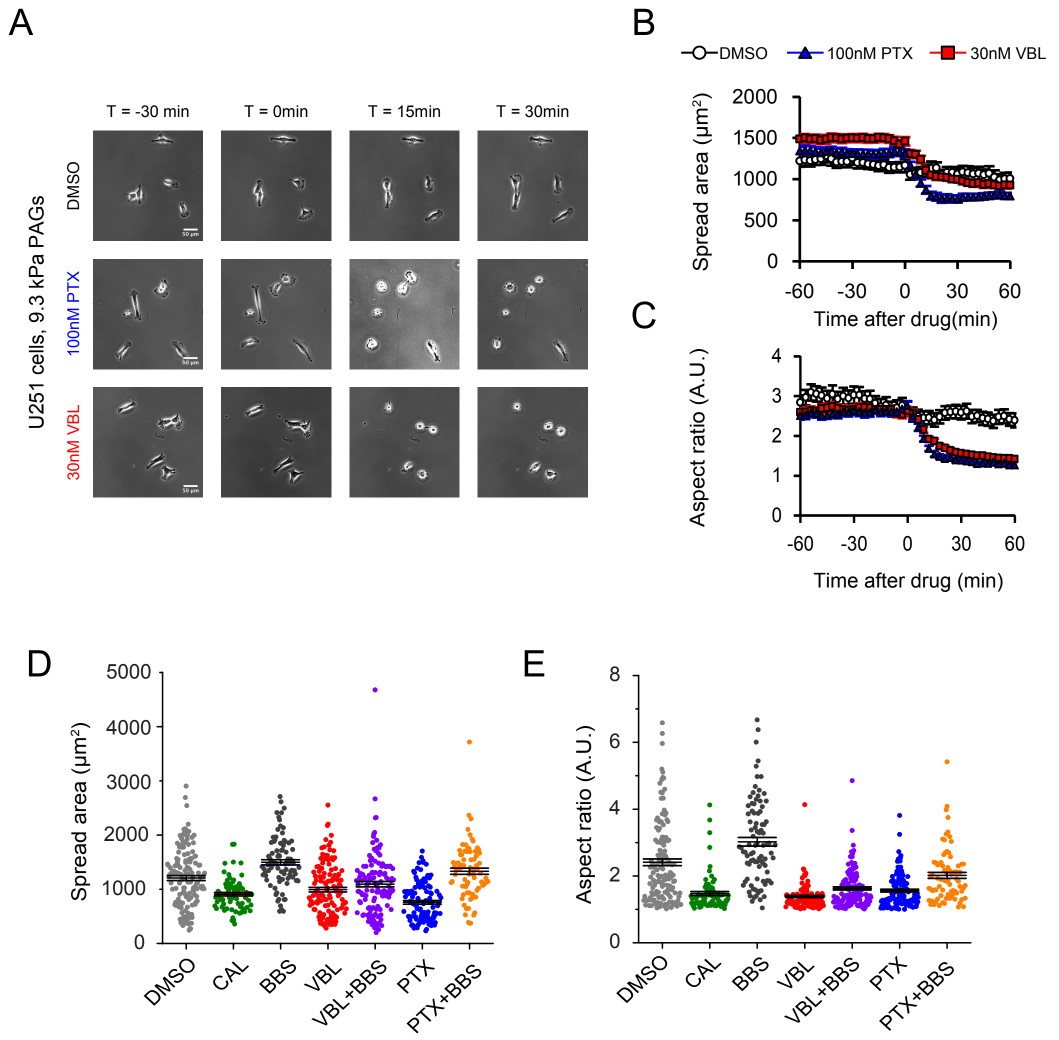

Figure S3. Effects of myosin II activators or inhibitors and MTAs on cell spreading and aspect ratio. Related to **Figures 2** and **4.**

(A) Time-lapse images of U251 cells treated with DMSO, 100 nM PTX, or 30 nM VBL. Images acquired every 3 minutes at 10x magnification using phase contrast optics. Time represents time after drug addition (at T = 0). Scale, 50 µm

(B) Spread area measurements before and after addition of drug for the conditions in panel C; n = 179, 127, 108 cells.

(C) Quantification of aspect ratio for the same cells in panel C. Error bars represent mean ± SEM.

(D) Quantification of U251 cell spread area on 9.3 kPa PAGs. Cells were treated with media containing DMSO or drugs and images were acquired 30 minutes post-treatment. Treatment conditions: DMSO (vehicle control), CAL (1 nM), BBS (10 µM), VBL (30 nM), VBL + BBS (30 nM VBL and 10 µM BBS), PTX

(100 nM), or PTX + BBS (100 nM PTX + 10 μM BBS), n = 179, 78, 90, 108, 133, 127, 79 cells. DMSO, PTX, and VBL data obtained at 30 minutes from the data in panels B and C.

(E) Quantification of aspect ratio for the conditions shown in panel D. Error bars represent mean ± SEM, pairwise statistics are reported in **Table S2**.

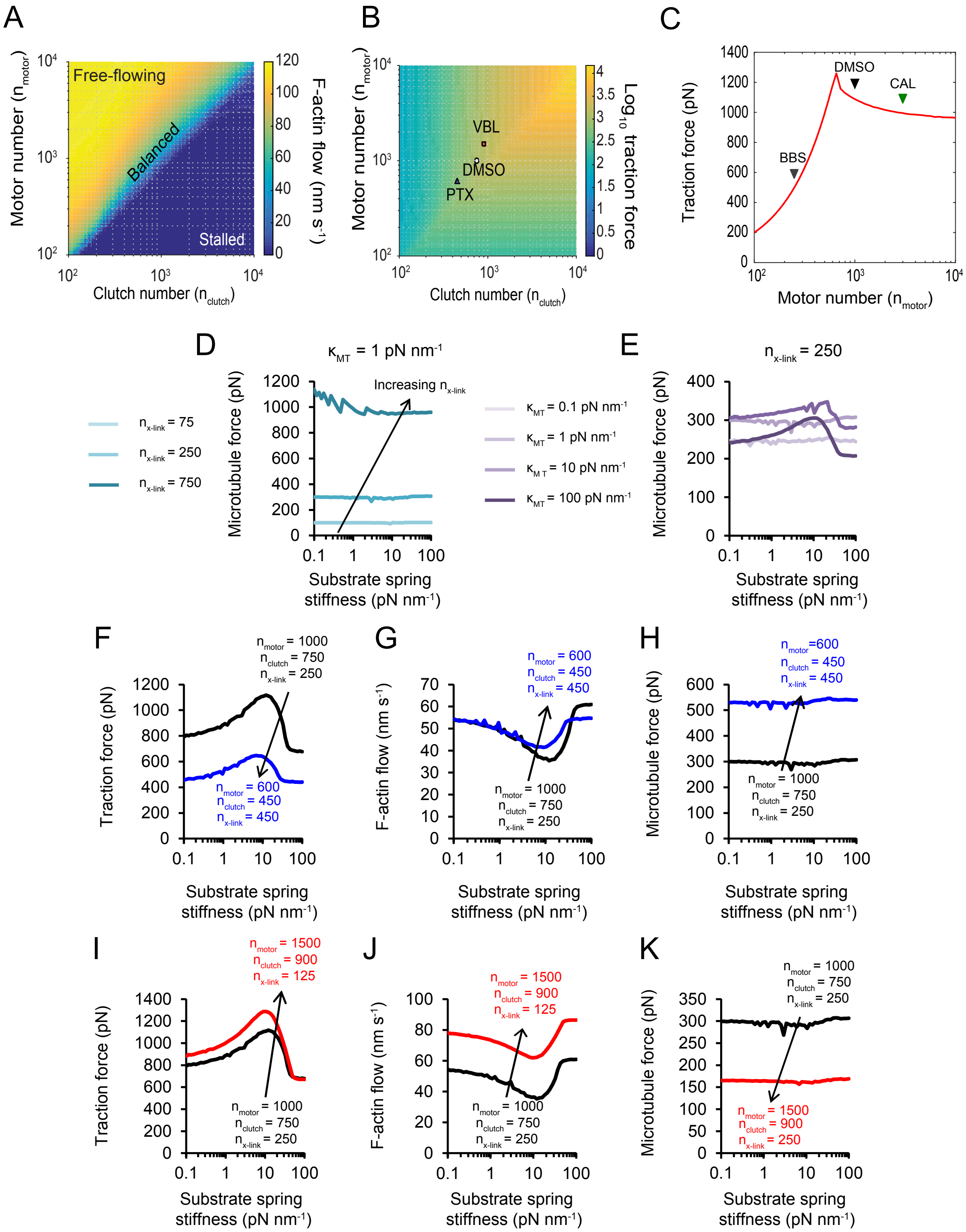

A
Free-flowing
Balanced
Stalled
Motor number (n_motor)
Clutch number (n_clutch)
F-actin flow (nm s-1)
B
VBL
DMSO
PTX
Motor number (n_motor)
Clutch number (n_clutch)
Log10 traction force
C
DMSO
CAL
BBS
Traction force (pN)
Motor number (n_motor)
D
κMT = 1 pN nm-1
Increasing n_x-link
n_x-link = 75
n_x-link = 250
n_x-link = 750
Microtubule force (pN)
Substrate spring stiffness (pN nm-1)
E
n_x-link = 250
κMT = 0.1 pN nm-1
κMT = 1 pN nm-1
κMT = 10 pN nm-1
κMT = 100 pN nm-1
Microtubule force (pN)
Substrate spring stiffness (pN nm-1)
F
n_motor = 1000
n_clutch = 750
n_x-link = 250
n_motor = 600
n_clutch = 450
n_x-link = 450
Traction force (pN)
Substrate spring stiffness (pN nm-1)
G
n_motor = 600
n_clutch = 450
n_x-link = 450
n_motor = 1000
n_clutch = 750
n_x-link = 250
F-actin flow (nm s-1)
Substrate spring stiffness (pN nm-1)
H
n_motor =600
n_clutch = 450
n_x-link = 450
n_motor = 1000
n_clutch = 750
n_x-link = 250
Microtubule force (pN)
Substrate spring stiffness (pN nm-1)
I
n_motor = 1500
n_clutch = 900
n_x-link = 125
n_motor = 1000
n_clutch = 750
n_x-link = 250
Traction force (pN)
Substrate spring stiffness (pN nm-1)
J
n_motor = 1500
n_clutch = 900
n_x-link = 125
n_motor = 1000
n_clutch = 750
n_x-link = 250
F-actin flow (nm s-1)
Substrate spring stiffness (pN nm-1)
K
n_motor = 1000
n_clutch = 750
n_x-link = 250
n_motor = 1500
n_clutch = 900
n_x-link = 250
Microtubule force (pN)
Substrate spring stiffness (pN nm-1)

Figure S4. Motor-clutch phase space plots to explain effects of MTAs and drugs that influence myosin II activity. Related to **Figure 3** and **4**.

(A) Phase space plot of motor-clutch model output actin flow speed as a function of n_{motor} and n_{clutch}. Balanced represents the region $n_{motor} = n_{clutch}$ ($N_{mc} = 1$). Free-flowing systems have $n_{motor}>n_{clutch}$ ($N_{mc}>1$) and flows near the maximal unloaded speed (v_{motor} = 120nm s^{-1}). Stalled systems have $n_{motor}<n_{clutch}$ ($N_{mc}<1$) and near-zero flows.

(B) Phase space plot of log_{10}-transformed traction force as a function of n_{motor} and n_{clutch}. Coordinate increases in n_{motor} and n_{clutch} increase the total traction force output. Circles represent the simulation parameters for DMSO (white), PTX (blue), and VBL (red).

(C) Contour of traction force as a function of n_{motor} for a fixed number of clutches (n_{clutch} = 750). Parameter space estimates for DMSO, calyculin A (CAL), and blebbistatin (BBS), are shown corresponding to simulations in **Figure 4C**. Note that force decreases weakly as motor number increases (e.g. upon treatment with CAL), and much more strongly as motor number decreases past the optimum at $N_{mc}\approx1$ (e.g. upon treatment with BBS). All simulations in panels A-C were run at κ_{sub} = 10 pN nm^{-1}.

(D) Force on the microtubule spring for simulation conditions in **Figure 5B** and **C**, where $n_{x\text{-}link}$ was varied as an independent parameter; $n_{x\text{-}link}$ = 0 (black), 75, 250, and 750.

(E) Force on the microtubule spring for simulation conditions in **Figure 5D** and **E**, where κ_{MT} was varied as an independent parameter; κ_{MT} = 0.1, 1, 10, and 100 pN nm^{-1}.

(F-H) Model-predicted traction force and F-actin flow from simulations where n_{motor}, n_{clutch}, and $n_{x\text{-}link}$ were varied to reproduce experimental trends in cells treated with DMSO or PTX.

(I-K) Model-predicted traction force and F-actin flow from simulations where n_{motor}, n_{clutch}, and $n_{x\text{-}link}$ were varied to reproduce experimental trends in cells treated with DMSO or VBL. All other simulation parameters are in **Methods S1**.

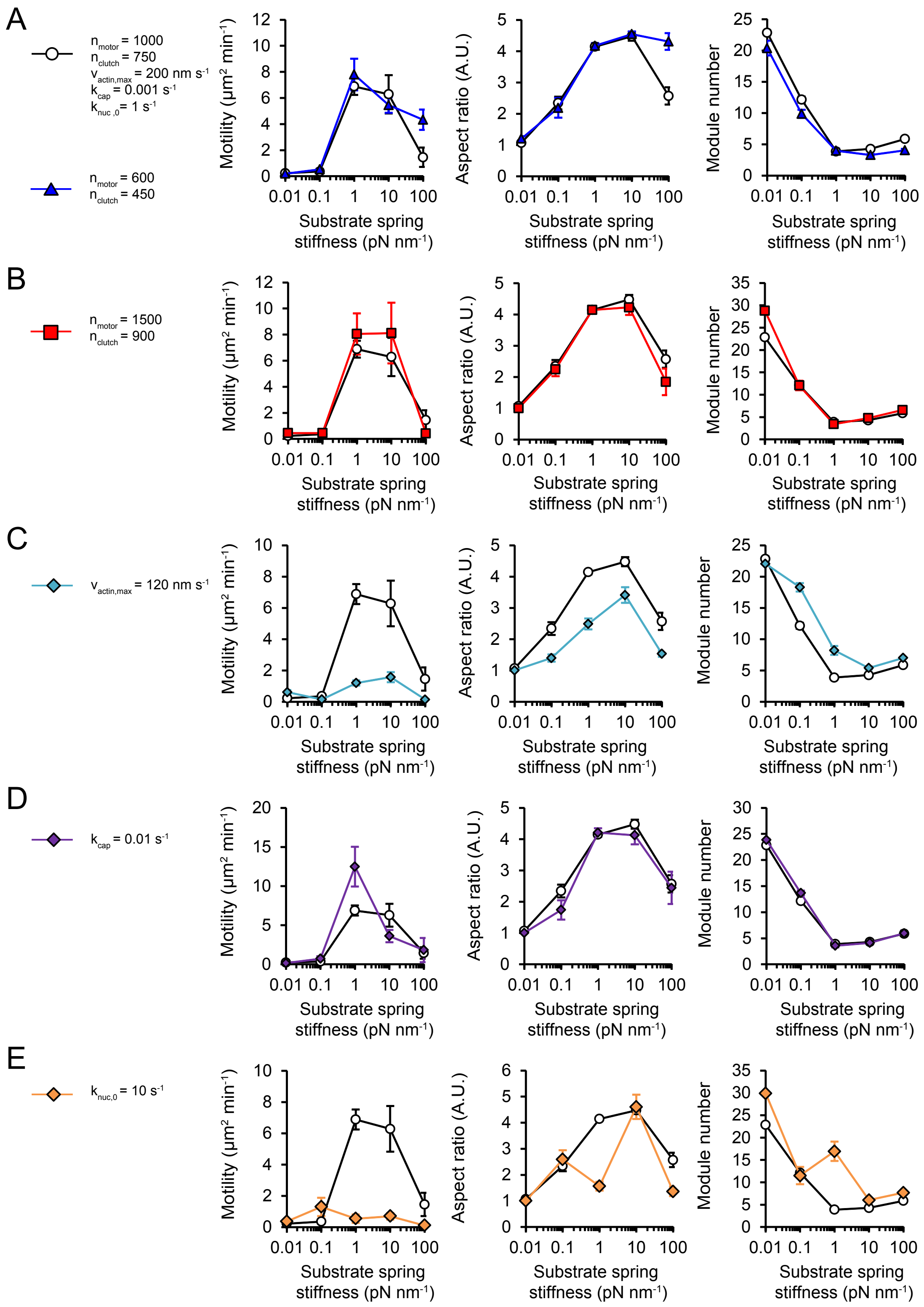

A
n_{motor} = 1000
n_{clutch} = 750
$v_{actin,max}$ = 200 nm s^{-1}
k_{cap} = 0.001 s^{-1}
$k_{nuc,0}$ = 1 s^{-1}
n_{motor} = 600
n_{clutch} = 450
B
n_{motor} = 1500
n_{clutch} = 900
C
$v_{actin,max}$ = 120 nm s^{-1}
D
k_{cap} = 0.01 s^{-1}
E
$k_{nuc,0}$ = 10 s^{-1}
Motility (µm² min^{-1})
Aspect ratio (A.U.)
Module number
Substrate spring stiffness (pN nm^{-1})
0.01
0.1
1
10
100

Figure S5. Simulated cell migration and shape for MTA parameter sets and simulations with altered protrusion dynamics. Related to **Figure 6**.

(A) Motility coefficient, aspect ratio, and module number for CMS runs using reference parameters (white circles; n = 14, 8, 36, 48, 24 runs) or a PTX parameter set (blue triangles; n = 8, 8, 16, 24, 20 runs).

(B) Motility coefficient, aspect ratio, and module number for CMS runs using reference parameters or a VBL parameter set (red squares; n = 4, 7, 8, 8, 8 runs).

(C) Motility coefficient, aspect ratio, and module number for CMS runs using reference parameters or simulations where $v_{actin,max}$ = 120 nm s^{-1} (cyan diamonds; n = 7, 14, 22, 30, 16 runs).

(D) Motility coefficient, aspect ratio, and module number for CMS runs using reference parameters or simulations where k_{cap} = 0.01 s^{-1} (purple diamonds; n = 7, 8, 8, 16, 8 runs).

(E) Motility coefficient, aspect ratio, and module number for CMS runs using using reference parameters or simulations where k_{nuc} = 10 s^{-1} (orange diamonds; n = 16, 8, 12, 8, 8 runs). Reference parameter simulation data are the same as in **Figure 6** and **Figure S6** and reported in **Methods S1**. Data are represented as mean ± SEM, pairwise statistics for motility are in **Table S3**.

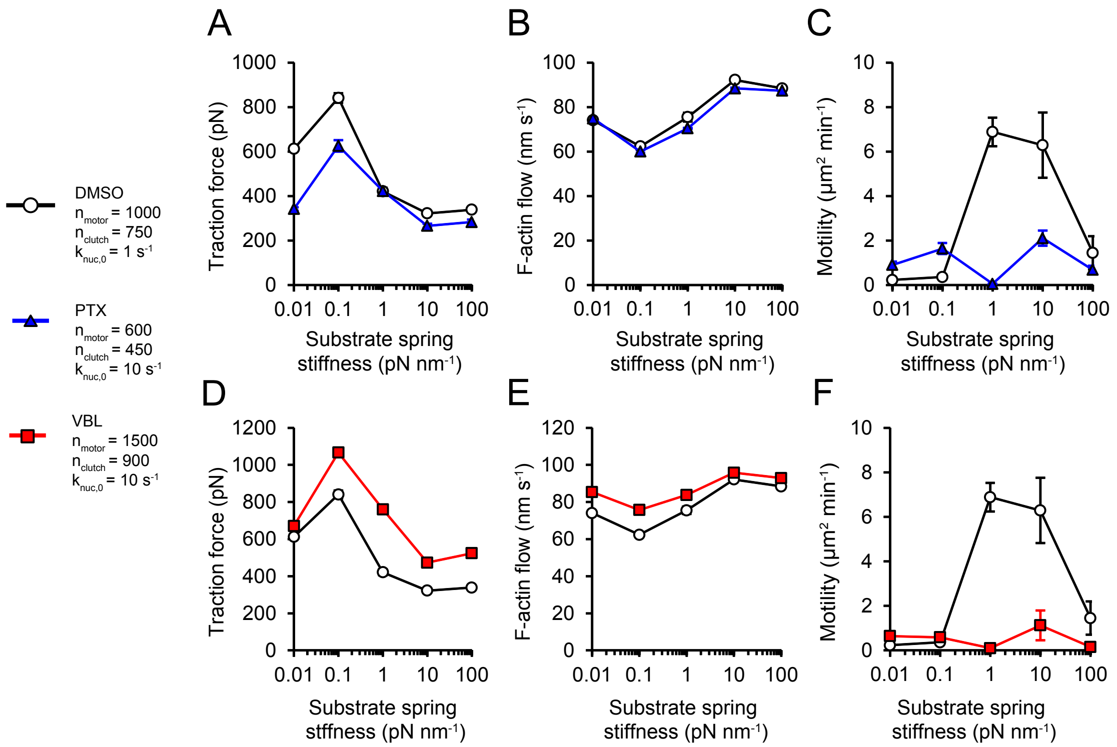

Figure S6. Alternative parameter sets for simulated MTA-treated simulated cells. Related to **Figure 6**.

(A-C) CMS predictions of traction force, F-actin flow, and motility for reference parameters (white circles; n = 14, 8, 36, 48, 24 runs) or a PTX parameter set (blue triangles; n = 8, 12, 8, 8, 12 runs).

(D-F) CMS predictions of traction force, F-actin flow, and motility for reference parameters (repeated from panels A-C) or a VBL parameter set (red squares; VBL: n = 12 runs at each stiffness). Reference parameter simulation data are the same as in **Figure 6** and **Figure S5** and reported in **Methods S1**. Data are represented as mean ± SEM, pairwise statistics for motility are in **Table S3**.

Supplemental tables

Table S1. Pairwise statistical tests of spread area, aspect ratio, motility, strain energy, and F-actin flow speed in vehicle and MTA-treated U251 cells. Related to **Figure 2** and **Figure 3**.

	PAG Young's modulus (kPa)					
	0.7 kPa	4.6 kPa	9.3 kPa	20 kPa	98 kPa	193 kPa
**Figure 2**. Spread area						
DMSO vs 1 nM PTX	0.11		10^{-3}		10^{-4}	10^{-12}
DMSO vs 100 nM PTX	10^{-12}		10^{-13}		**	10^{-15}
1 nM PTX vs 100 nM PTX	10^{-5}		10^{-4}		10^{-9}	0.61
DMSO vs 3 nM VBL	0.30		0.84		10^{-6}	10^{-10}
DMSO vs 30 nM VBL	10^{-9}		10^{-3}		10^{-8}	0.93
3 nM VBL vs 30 nM VBL	10^{-3}		10^{-4}		0.99	10^{-7}
**Figure 2**. Aspect ratio						
DMSO vs 1 nM PTX	0.82		0.12		0.026	10^{-11}
DMSO vs 100 nM PTX	**		**		**	**
1 nM PTX vs 100 nM PTX	10^{-12}		10^{-13}		10^{-15}	0.013
DMSO vs 3 nM VBL	10^{-7}		10^{-5}		10^{-15}	**
DMSO vs 30 nM VBL	**		**		**	10^{-10}
3 nM VBL vs 30 nM VBL	0.12		10^{-5}		0.038	0.085
**Figure 2**. Motility						
DMSO vs 1 nM PTX	0.98		10^{-4}		10^{-6}	0.011
DMSO vs 100 nM PTX	10^{-7}		10^{-11}		10^{-12}	10^{-8}
1 nM PTX vs 100 nM PTX	10^{-5}		0.041		0.45	0.024
DMSO vs 3 nM VBL	10^{-3}		10^{-3}		10^{-12}	10^{-3}
DMSO vs 30 nM VBL	10^{-3}		10^{-6}		**	10^{-7}
3 nM VBL vs 30 nM VBL	0.99		0.95		0.96	0.16
**Figure 3**. Strain energy						
DMSO vs 100 nM PTX	0.99	0.99	10^{-6}	10^{-3}		
DMSO vs 30 nM VBL	0.21	0.31	0.99	10^{-11}		
100 nM PTX vs 30 nM VBL	0.29	0.58	10^{-5}	10^{-3}		
**Figure 3**. Actin flow						
DMSO vs 100 nM PTX	0.062	0.11	0.11	10^{-5}	0.99	
DMSO vs 30 nM VBL	0.99	10^{-6}	10^{-6}	0.18	0.69	
100 nM PTX vs 30 nM VBL	0.15	0.012	0.012	10^{-7}	0.66	

Groups were compared using a Kruskal-Wallis test with Dunn-Sidăk correction for multiple comparisons. Shading indicates statistical significance at $p < 0.05$ (**, $p < 10^{-15}$), tests resulting in $p < 10^{-3}$ are rounded to the nearest decimal.

Table S2. Pairwise statistical comparisons of strain energy, spread area, or aspect ratio in U251 cells treated with MTAs, myosin II drugs, or both. Related to **Figure 4** and **Figure S3**.

Figure 4B
Strain Energy

	CAL	BBS	VBL	VBL+BBS	PTX	PTX+BBS
DMSO	0.76	10^{-11}	0.99	10^{-5}	0.0013	10^{-9}
CAL		0.0074	0.0074	0.28	0.99	0.008
BBS			10^{-9}	0.99	0.09	0.99
VBL				10^{-4}	0.0037	10^{-7}
VBL+BBS					0.92	0.98
PTX						0.067
PTX+BBS						

Figure S3A
Spread Area

	CAL	BBS	VBL	VBL+BBS	PTX	PTX+BBS
DMSO	10^{-3}	10^{-4}	10^{-3}	0.58	10^{-11}	0.72
CAL		10^{-12}	0.99	0.29	0.49	10^{-6}
BBS			10^{-11}	10^{-8}	**	0.59
VBL				0.97	0.0047	10^{-5}
VBL+BBS					10^{-5}	0.0067
PTX						10^{-3}
PTX+BBS						

Figure S3B
Aspect Ratio

	CAL	BBS	VBL	VBL+BBS	PTX	PTX+BBS
DMSO	10^{-11}	0.0017	**	10^{-7}	10^{-9}	0.99
CAL		**	1	0.3	0.95	10^{-6}
BBS			**	**	**	10^{-4}
VBL				0.021	0.36	10^{-8}
VBL+BBS					0.99	0.0073
PTX						10^{-9}
PTX+BBS						

Shading indicates statistical significance at $p < 0.05$ (**, $p < 10^{-15}$). Tests resulting in $p < 10^{-3}$ are rounded to the nearest decimal. Pairwise p-values obtained using a Kruskal-Wallis test with Dunn-Sidăk correction for multiple comparisons.

Table S3. Pairwise p-values for motility compared to reference parameters. Related to **Figure 6**, **S5**, and **S6**.

	Substrate Spring Constant (pN nm^{-1})				
	0.01	0.1	1	10	100
PTX with reduced $v_{actin,max}$ (Figure 6B-D) *n_{motor} = 600, n_{clutch} = 450, v_{actin} = 120nm $^{-1}$*	0.0051	0.18	10^{-7}	0.59	0.034
VBL with reduced $v_{actin,max}$ (Figure 6E-G) *n_{motor} = 1500, n_{clutch} = 900, v_{actin} = 120nm s^{-1}*	0.0029	0.25	10^{-8}	0.036	0.24
PTX motor/clutch only (Figure S5A) *n_{motor} = 600, n_{clutch} = 450*	0.38	0.046	0.61	0.045	10^{-4}
VBL motor/clutch only (Figure S5B) *n_{motor} = 1500, n_{clutch} = 900*	0.034	0.91	0.62	0.21	0.69
Reduced v_{actin} (Figure S5C) *$v_{actin,max}$ = 120 nm s^{-1}*	0.0023	0.0027	10^{-7}	10^{-4}	0.077
Increased k_{cap} (Figure S5D) *k_{cap} = 0.01 s^{-1}*	0.12	0.25	0.022	0.39	0.70
Increased k_{nuc} (Figure S5E) *$k_{nuc,0}$ = 1 s^{-1}*	0.060	0.25	10^{-6}	10^{-3}	0.030
PTX with increased $k_{nuc,0}$ (Figure S6A-C) *n_{motor} = 600, n_{clutch} = 450, $k_{nuc,0}$ = 10 s^{-1}*	10^{-3}	10^{-3}	10^{-6}	0.048	0.12
VBL with increased $k_{nuc,0}$ (Figure S7D-F) *n_{motor} = 1500, n_{clutch} = 900, $k_{nuc,0}$ = 10 s^{-1}*	0.0033	0.54	10^{-4}	0.0050	0.087

Pairwise p-values were obtained at each substrate spring constant value using a Kruskal-Wallis test. Shading indicates statistical significance at $p < 0.05$. Tests resulting in $p < 10^{-3}$ are rounded to the nearest decimal.

Method Details

Methods S1. Motor-clutch model and CMS reference parameter sets, Related to **Figures 3**, **5**, **6**, and **S4-S6.**

Parameter	Description	Range
Motor parameters		
n_{motor}	Number of myosin II motors	1,000
F_{motor}	Myosin II stall force	2 pN
v_{motor}	Unloaded (maximum) myosin II velocity	120 nm s^{-1}
Clutch parameters		
n_{clutch}	Number of molecular clutches	750
F_{bond}	Characteristic slip bond force for clutches	2 pN
κ_{clutch}	Clutch spring stiffness	0.8 pN nm^{-1}
k_{on}	Pseudo-first order binding rate for clutches to F-actin	1 s^{-1}
k_{off}	Basal first-order clutch unbinding rate	0.1 s^{-1}
SubstratepParameters		
κ_{sub}	Substrate spring stiffness	0.01-100 pN nm^{-1}
*Microtubule parameters (**Figure 5** and **Figure S5**)*		
κ_{MT}	Microtubule stiffness	1 pN nm^{-1}
$n_{x = link}$	Number of F-actin-microtubule cross-linkers	75-750
$F_{bond,x\text{-}link}$	Characteristic slip bond force for cross-linkers	2 pN
$\kappa_{x\text{-}link}$	Cross-linker spring stiffness	0.8 pN nm^{-1}
$k_{on,x\text{-}link}$	Pseudo-first order binding rate for cross-linkers to F-actin	1 s^{-1}
$k_{off,x\text{-}link}$	Basal first-order cross-linker unbinding rate	0.1 s^{-1}
*Cell migration simulator parameters (**Figure 6** and **Figure S6-7**)*		
k_{cap}	Module capping rate	0.001 s^{-1}
$k_{nuc,0}$	Maximum module nucleation rate	1 s^{-1}
$v_{actin,max}$	Maximum actin polymerization velocity	200 nm s^{-1}
A_{total}	Total actin pool available for protrusions	100 µm
κ_{cell}	Cell spring constant	10^4 pN nm^{-1}
L_{cell}	Initial module length	5 µm
L_{min}	Minimum module length	100 nm
$n_{clutch,cell}$	Number of cell body clutches	10
